# Supplementary material for: Population-specific causal disease effect sizes in functionally important regions impacted by selection
Source: Nat Commun. 2021 Feb 17;12:1098. doi: 10.1038/s41467-021-21286-1 (PMC7889654; doi:10.1038/s41467-021-21286-1)
Supplement: Supplementary file 3 — Reporting Summary [file 41467_2021_21286_MOESM3_ESM.pdf]

nature research

Corresponding author(s): Huwenbo Shi

Last updated by author(s): Dec 21, 2020

Reporting Summary

Nature Research wishes to improve the reproducibility of the work that we publish. This form provides structure for consistency and transparency in reporting. For further information on Nature Research policies, see our Editorial Policies and the Editorial Policy Checklist.

Statistics

For all statistical analyses, confirm that the following items are present in the figure legend, table legend, main text, or Methods section.

n/a	Confirmed	
☐	☒	The exact sample size (n) for each experimental group/condition, given as a discrete number and unit of measurement
☐	☒	A statement on whether measurements were taken from distinct samples or whether the same sample was measured repeatedly
☐	☒	The statistical test(s) used AND whether they are one- or two-sided<br>*Only common tests should be described solely by name; describe more complex techniques in the Methods section.*
☒	☐	A description of all covariates tested
☐	☒	A description of any assumptions or corrections, such as tests of normality and adjustment for multiple comparisons
☐	☒	A full description of the statistical parameters including central tendency (e.g. means) or other basic estimates (e.g. regression coefficient) AND variation (e.g. standard deviation) or associated estimates of uncertainty (e.g. confidence intervals)
☐	☒	For null hypothesis testing, the test statistic (e.g. F, t, r) with confidence intervals, effect sizes, degrees of freedom and P value noted<br>*Give P values as exact values whenever suitable.*
☒	☐	For Bayesian analysis, information on the choice of priors and Markov chain Monte Carlo settings
☒	☐	For hierarchical and complex designs, identification of the appropriate level for tests and full reporting of outcomes
☐	☒	Estimates of effect sizes (e.g. Cohen's d, Pearson's r), indicating how they were calculated

Our web collection on statistics for biologists contains articles on many of the points above.

Software and code

Policy information about availability of computer code

Data collection	We did not use any software to collect data.
Data analysis	Python code implementing S-LDXR (version 0.3-beta) is available at (https://github.com/huwenboshi/s-ldxr).<br>Python code for simulating GWAS summary statistics under the baseline-LD-X model is available at (https://github.com/huwenboshi/s-ldxr-sim).<br>Python code implementing the two-population Eyre-Walker model is available at (https://github.com/huwenboshi/two-population-Eyre-Walker-model).<br>Python code for creating the distance to nearest exon annotation is available at (https://github.com/huwenboshi/distance-to-nearest-exon}).<br>We used HAPGEN2 (https://mathgen.stats.ox.ac.uk/genetics_software/hapgen/hapgen2.html} to simulated genotype data.<br>We used PLINK2 (https://www.cog-genomics.org/plink/2.0/) to remove related individuals in the simulated genotype data.

For manuscripts utilizing custom algorithms or software that are central to the research but not yet described in published literature, software must be made available to editors and reviewers. We strongly encourage code deposition in a community repository (e.g. GitHub). See the Nature Research guidelines for submitting code & software for further information.

Data

Policy information about availability of data

All manuscripts must include a data availability statement. This statement should provide the following information, where applicable:

- Accession codes, unique identifiers, or web links for publicly available datasets
- A list of figures that have associated raw data
- A description of any restrictions on data availability

All baseline-LD-X model annotations and other annotations used in this work are available at (https://data.broadinstitute.org/alkesgroup/S-LDXR/).

We used exon definitions from the UCSC Genome Browser (https://genome.ucsc.edu/).
We used gene pLI scores from the Exome Aggregation Consortium (ExAC) (https://exac.broadinstitute.org/).
The integrated haplotype scores (iHS) are available at (http://coruscant.itmat.upenn.edu/data/JohnsonEA_iHSscores.tar.gz).
The 1000 Genomes Project Phase 3 data are available at (https://www.internationalgenome.org/).
The baseline-LD model annotations are available at (https://alkesgroup.broadinstitute.org/LDSCORE/).

Field-specific reporting

Please select the one below that is the best fit for your research. If you are not sure, read the appropriate sections before making your selection.

- [x] Life sciences
- [ ] Behavioural & social sciences
- [ ] Ecological, evolutionary & environmental sciences

For a reference copy of the document with all sections, see nature.com/documents/nr-reporting-summary-flat.pdf

Life sciences study design

All studies must disclose on these points even when the disclosure is negative.

Sample size	We used GWAS sample size as reported in the relevant publications in our analysis. For example, we used the sample size reported in Loh et al. 2018 Nature Genetics to determine the sample size of UK Biobank GWAS summary statistics. And we used the sample size reported in Kanai et al. 2018 Nature Genetics to determine the sample size of Biobank Japan GWAS summary statistics.
Data exclusions	We excluded from our analysis diseases and complex traits that are not heritable. This is appropriate as trans-ethnic genetic correlation is not defined for traits that are not heritable.
Replication	We did not perform explicit replication by performing independent GWAS studies, which would require a large amount of effort. However, we replicated our results by restricting our meta-analysis to a subset of independent diseases and complex traits.
Randomization	We did not collect data or perform any randomization by ourselves. This is because our study relies on published GWAS summary statistics data.
Blinding	We did not collect data or perform any blinding by ourselves. This is because our study relies on published GWAS summary statistics data.

Reporting for specific materials, systems and methods

We require information from authors about some types of materials, experimental systems and methods used in many studies. Here, indicate whether each material, system or method listed is relevant to your study. If you are not sure if a list item applies to your research, read the appropriate section before selecting a response.

Materials & experimental systems

n/a	Involved in the study	
x		Antibodies
x		Eukaryotic cell lines
x		Palaeontology and archaeology
x		Animals and other organisms
x		Human research participants
x		Clinical data
x		Dual use research of concern

Methods

n/a	Involved in the study	
x		ChIP-seq
x		Flow cytometry
x		MRI-based neuroimaging
