# Supplementary material for: Population-specific causal disease effect sizes in functionally important regions impacted by selection
Source: Nat Commun. 2021 Feb 17;12:1098. doi: 10.1038/s41467-021-21286-1 (PMC7889654; doi:10.1038/s41467-021-21286-1)
Supplement: Supplementary file 4 — Description of Additional Supplementary Files [file 41467_2021_21286_MOESM4_ESM.pdf]

Description of Additional Supplementary Files

Supplementary Data 1

List of baseline-LD-X model annotations. **a**) Summary information for functional annotations. **b**) Summary information for continuous-valued annotations. **c**) Correlation between functional and continuous-valued annotations. **d**) Correlation between continuous-valued annotations.

Supplementary Data 2

List of the specifically expressed gene (SEG) annotations. **a**) Mean background selection statistic of SEG annotations. **b**) Correlation between SEG annotation and continuous-valued annotations.

Supplementary Data 3

Numerical results of S-LDXR in null simulations with 1% causal SNPs. The shrinkage parameter, α, is set to 0.0 in **a**, 0.25 in **b**, 0.5 in **c**, 0.75 in **d**, and 1.0 in **e**. Positive rate is defined as the proportion of tests with p-value less than 0.05. Standard error of the positive rate is obtained using jackknife.

Supplementary Data 4

Numerical results of S-LDXR in null simulations with 10% causal SNPs. The shrinkage parameter, α, is set to 0.0 in **a**, 0.25 in **b**, 0.5 in **c**, 0.75 in **d**, and 1.0 in **e**. Positive rate is defined as the proportion of tests with p-value less than 0.05. Standard error of the positive rate is obtained using jackknife.

Supplementary Data 5

Numerical results of S-LDXR in null simulations with 100% causal SNPs. The shrinkage parameter, α, is set to 0.0 in **a**, 0.25 in **b**, 0.5 in **c**, 0.75 in **d**, and 1.0 in **e**. Positive rate is defined as the proportion of tests with p-value less than 0.05. Standard error of the positive rate is obtained using jackknife.

Supplementary Data 6

Numerical results of S-LDXR in causal simulations with 1% causal SNPs. The shrinkage parameter, α, is set to 0.0 in **a**, 0.25 in **b**, 0.5 in **c**, 0.75 in **d**, and 1.0 in **e**. Positive rate is defined as the proportion of tests with p-value less than 0.05. Standard error of the positive rate is obtained using jackknife.

Supplementary Data 7

Numerical results of S-LDXR in causal simulations with 10% causal SNPs. The shrinkage parameter, α, is set to 0.0 in **a**, 0.25 in **b**, 0.5 in **c**, 0.75 in **d**, and 1.0 in **e**. Positive rate is defined as the proportion of tests with p-value less than 0.05. Standard error of the positive rate is obtained using jackknife.

Supplementary Data 8

Numerical results of S-LDXR in causal simulations with 100% causal SNPs. The shrinkage parameter, α, is set to 0.0 in **a**, 0.25 in **b**, 0.5 in **c**, 0.75 in **d**, and 1.0 in **e**. Positive rate is defined as the proportion of tests with p-value less than 0.05. Standard error of the positive rate is obtained using jackknife.

Supplementary Data 9

Numerical results of S-LDXR in null simulations with annotation-dependent MAF-dependent genetic architectures. 10% of SNPs were randomly selected to be causal. The shrinkage parameter, α, is set to 0.0 in **a**, 0.25 in **b**, 0.5 in **c**, 0.75 in **d**, and 1.0 in **e**. Positive rate is defined as the proportion of tests with p-value less than 0.05. Standard error of the positive rate is obtained using jackknife.

Supplementary Data 10

Numerical results of S-LDXR in null simulations with annotation-dependent MAF-dependent genetic architectures and 5 MAF bins added to the baseline-LD-X model. 10% of SNPs were randomly selected to be causal. The shrinkage parameter, α, is set to 0.0 in **a**, 0.25 in **b**, 0.5 in **c**, 0.75 in **d**, and 1.0 in **e**. Positive rate is defined as the proportion of tests with p-value less than 0.05. Standard error of the positive rate is obtained using jackknife.

Supplementary Data 11

Numerical results of S-LDXR in null simulations where causal variants differ across the two populations. Here, 10% of SNPs were randomly selected to be causal in each population, with 80% of the causal variants in each population shared with the other population. The shrinkage parameter, α, is set to 0.0 in **a**, 0.25 in **b**, 0.5 in **c**, 0.75 in **d**, and 1.0 in **e**. Positive rate is defined as the proportion of tests with p-value less than 0.05. Standard error of the positive rate is obtained using jackknife.

Supplementary Data 12

Numerical results of S-LDXR in null simulations using all simulated GWAS samples and half (250) the default reference sample size. Here, 10% of SNPs were randomly selected to be causal. Shrinkage level, α, was set to 0.5. **a**) Estimates of $\lambda^2(C)$for binary annotations and quintiles of continuously valued annotation in simulations under the baseline-LD-X model. **b**) Estimates of $\lambda^2(C)$ for binary annotations and quintiles of continuously valued annotation in simulations under the model involving annotation-dependent and MAF-dependent genetic architectures. Mean and standard errors were obtained across 1,000 simulations.

Supplementary Data 13

Numerical results of S-LDXR in null simulations using all simulated GWAS samples and twice (1,000) the default reference sample size. Here, 10% of SNPs were randomly selected to be causal. Shrinkage level, α, was set to 0.5. **a**) Estimates of $\lambda^2(C)$for binary annotations and quintiles of continuously valued annotation in simulations under the baseline-LD-X model. **b**) Estimates of $\lambda^2(C)$ for binary annotations and quintiles of continuously valued annotation in simulations under the model involving annotation-dependent and MAF-dependent genetic architectures. Mean and standard errors were obtained across 1,000 simulations.

Supplementary Data 14

Numerical results of S-LDXR in null simulations using half of the simulated GWAS samples and default (500) reference sample size. Here, 10% of SNPs were randomly selected to be causal. Shrinkage level, α, was set to 0.5. **a**) Estimates of $\lambda^2(C)$ for binary annotations and quintiles of continuously valued annotation in simulations under the baseline-LD-X model. **b**) Estimates of $\lambda^2(C)$ for binary annotations and quintiles of continuously valued annotation in simulations under the model involving annotation-

dependent and MAF-dependent genetic architectures. Mean and standard errors were obtained across 1,000 simulations.

Supplementary Data 15

Numerical S-LDXR results for quintiles of 8 continuous-valued annotations across 31 diseases and complex traits. The shrinkage parameter, α, was set to 0.0 in **a**, 0.5 in **b**, and 1.0 in **c**. **d**) Here, results were meta-analyzed across a subset of 20 approximately independent traits with default shrinkage parameter (α=0.5).

Supplementary Data 16

Numerical S-LDXR results for quintiles of 20 binary functional annotations across 31 diseases and complex traits. The shrinkage parameter, α, was set to 0.0 in **a**, 0.5 in **b**, and 1.0 in **c**. **d**) Here, results were meta-analyzed across a subset of 20 approximately independent traits with default shrinkage parameter (α=0.5).

Supplementary Data 17

Numerical S-LDXR results of observed $\lambda^2(C)$ vs. expected $\lambda^2(C)$ based on 8 continuous-valued annotations for 20 binary annotations across 31 diseases and complex traits. The shrinkage parameter, α, was set to 0.0 in **a**, 0.5 in **b**, and 1.0 in **c**.

Supplementary Data 18

Numerical S-LDXR results for 53 specifically expressed gene (SEG) annotations across 31 diseases and complex traits. While heritability enrichment may be impacted by choice of diseases and traits, $\lambda^2(C)$ is not expected to be disease specific. The shrinkage parameter, α, was set to 0.0 in **a**, 0.5 in **b**, and 1.0 in **c**.

Supplementary Data 19

Numerical S-LDXR results for 53 specifically expressed gene (SEG) annotations for 14 blood-related traits vs. 17 other traits. The list of 14 blood phenotypes is: BASO, EO, HBA1C, HGB, HTC, LYMPH, MCH, MCHC, MCV, MONO, NEUT, PLT, RBC, WBC. The list of 17 non-blood phenotype is: AF, AMN, AMP, BMI, BS, DBP, EGFR, HEIGHT, HDL, LDL, MDD, RA, SBP, SCZ, TC, TG, T2D. Full name of the abbreviations can be found in Table S2. Here, the shrinkage parameter α was set to the default of 0.5.

Supplementary Data 20

Numerical S-LDXR results of observed $\lambda^2(C)$ vs. expected $\lambda^2(C)$ based on 8 continuous-valued annotations for 53 specifically expressed gene (SEG) annotations across 31 diseases and complex traits. While heritability enrichment may be impacted by choice of diseases and traits, $\lambda^2(C)$ is not expected to be disease specific. The shrinkage parameter, α, was set to 0.0 in **a**, 0.5 in **b**, and 1.0 in **c**.

Supplementary Data 21

Numerical S-LDXR results of $\lambda^2(C)$ for 53 specifically expressed gene (SEG) annotations for each of the 31 diseases and complex traits. The shrinkage parameter, α, was set to the default value of 0.5.
