# Supplementary material for: Population-specific causal disease effect sizes in functionally important regions impacted by selection
Source: Nat Commun. 2021 Feb 17;12:1098. doi: 10.1038/s41467-021-21286-1 (PMC7889654; doi:10.1038/s41467-021-21286-1)
Supplement: Supplementary file 2 — Peer Review File [file 41467_2021_21286_MOESM2_ESM.pdf]

Reviewers' comments:

Reviewer #1 (Remarks to the Author):

In this paper, the authors introduce a new member of their LDSC methodology family, called S-LDXR, to estimate the trans-ethnic genetic correlations stratified by genomic functional annotations using GWAS summary statistics. Building on two previous notable LDSC models: bivariate-LDSC for estimating genetic correlations between traits in one population, and S-LDSC for estimating per-SNP heritability enrichment in functional annotations, this method combines the merit of the two and allows the genetic correlations between different ethnic populations to be variable across functional annotations, taking the population-specific LD into account. The authors apply the method on 30 diseases and complex traits in East Asians (EAS) and Europeans (EUR) with established genomic and tissue-specific annotations, in attempt to understand the biology and evolutionary cause underlying population-specific causal effect sizes.

Overall, I find the paper to be quite good and very well written. The methodology is solid with a lot of details and the results are very interesting. The authors conclude that strong GxE interaction at regions undergone positive selection is the most likely mechanism to give rise to population-specific causal effect sizes. While it is plausible, I have following concerns/questions to be addressed before I can fully agree. I hope the authors will find my comments useful.

Major:

1. The authors claim that the squared trans-ethnic genetic correlation (rg^2) is defined at causal effect sizes, so any depletion in lambda^2(C) = rg^2(C)/rg^2 observed for a functional category (C) must be due to the differences in causal effect sizes between the two ethnic populations. It is a bit hard for me to really believe that their rg^2 captures all causal effects, given that only HapMap3 common SNPs (MAF>5%) are included in the analysis and the various limitations in genotyping and imputation process. So, it is still a question for me that if a depletion in lambda^2(C) is due to the differences in causal effect sizes or due to the differences in LD and allele frequencies of the unobserved causal variants. This problem may become bigger when the causal variants with large effect sizes tend to be rarer in one population than in the other population due to differential actions of negative selection. Additionally, unlike S-LDSC, the S-LDXR does not include MAF bins as annotations, and the previous work has shown that the heritability estimate may be biased without stratifying SNPs by MAF, especially when the causal variants are enriched in low MAF bin. Thus, I suggest to investigate the unbiasness of lambda^2(C) estimate, as compared to that at the causal effects, by simulations that accounts for the properties of rare causal variants. For example, they can consider a model of negative selection where causal effect sizes are inversely related to MAF (with the relationship they found in Schoech et al 2019) and vary the proportion of causal variants being rare across functional categories.

2. In their simulation study for assessing the estimation of rg and lambda^2(C), they vary the true rg from 0.20 to 0.96, which is a bit odd. How about rg = 0 and rg = 1? The estimation performance at the extreme values of rg is important as there is a high interest especially in testing for rg = 1. Moreover, the EAS and EUR populations have roughly equal GWAS sample sizes in the simulation, which are actually very different in practice. The authors should simulate different sample sizes with a ratio similar to that in real data.

3. The power to detect enrichment or depletion in a binary annotation seems to be quite variable across different functional annotations (Figure 1b). While the reduction of power does not appear to be related to the annotation size, I wonder what factors affect the power, which could be important to understand any confounding factors in the results of real data analysis. For example, is the power related to the average LD score in the annotation?

4. They find that regions with strong depletion in lambda^2(C) are those with prior evidence of positive selection (coding, conserved, regulatory regions), and GxE interaction is a plausible mechanism for the differences in effect sizes across environments, therefore conclude that GxE interaction together with the influence of positive selection have resulted in causal effect sizes to be different across populations. However, these functional regions with lambda^2(C) depletion have also been found to have signatures of negative selection (especially conserved region). Related to my comment #1, the effect sizes at the common SNPs may appear to be different if the rare (or low-frequency) causal variant effect sizes are large and the LD between the rare variants and common SNPs are different between populations. In addition, the depletions of lambda^2(C) in the top quintile of background selection statistic and CpG content as well as the bottom quintile of nucleotide diversity and recombination rate (Figure 2b) seem to be more relevant to the model of negative selection. It seems the hypothesis of negative selection even without GxE can explain these results as well. How to reconcile these two hypotheses?

5. In Figure 2b, most annotations show a pattern that the enrichment or depletion of genetic correlation (lambda^2(C)) tends to be negatively related to that of heritability (h^2(g)) across quintiles. According to the argument in the paper, this could be the result of a combination of stronger GxE interaction and positive selection at functionally important regions (therefore higher per-SNP heritability). Then I find it is hard to interpret the clearly opposite patterns observed in the average LLD and GERP (NS), i.e. lambda^2(C) is positively related to h^2(C). The authors briefly mention it in the results but I find the explanation is a bit confusing.

6. In the Supplementary Note, they have a section describing a two-population Eyre-Walker model and find that a result similar to the real data analysis can be observed only when the fitness effects for deleterious SNPs differ in both mean and variance across populations. I find this to be interesting. If this is true, we can expect that the relationship between effect sizes and MAF will be different in the two populations. Given that there are methods available to estimate such a relationship using GWAS summary data (Gazal et al 2018 NG; Speed et al 2019 bioRxiv; Zeng et al 2019 bioRxiv), it would be interesting to test that in their data.

7. Besides SNP-specific Fst, have they looked at other per-SNP positive selection annotations, especially those for detecting recent selection?

Minor:

Line 90: It is not clear what C and C′ refer to and why C′ is needed here.

Why they estimate lambda^2(C) for binary annotations only or quintiles of continuous-valued annotations? Is there a technical difficulty to estimate that for a continuous annotation as a whole?

Line 419: As they define beta as per-allele effect size, the sum of per-SNP effect variance is not per-SNP heritability unless multiply by heterozygosity 2pq.

Figure S4 legend: panel # are incorrectly cited. Also, in "Shrinkage level, α, was set to 0.75 in a", is the number 0.75 correct? I thought it would only make sense if alpha is less than 0.5. Otherwise, the pattern does not seem to be coherent with Figure 1a, where alpha is 0.5 by default.

Figure S5 legend: the difference between a/b and c/d is the later includes a flanking window of 500bp for each annotation?

Line 450: tau_1C and tau_2C.

Reviewer #2 (Remarks to the Author):

The authors consider the problem of estimating genome-wide trans-ethnic genetic correlations. A new method named S-LDXR is proposed to measure trans-ethnic correlations for SNPs in different functional annotation categories. The S-LDXR method incorporates summary statistics from genome-wide association studies (GWAS) and linkage disequilibrium (LD) measures from reference panels The goal of S-LDXR is to identify functional annotation categories that have enrichment or depletion of squared trans-ethnic genetic correlation, where enrichment or depletion is calculated relative to genome-wide average genetic correlation. S-LDXR was evaluated in simulations studies using genotypes that were simulated based on haplotypes from East Asian and European populations. The authors find that estimated genome-wide average correlations for the two simulated populations were approximately unbiased for the simulated true values across different functional categories, and that S-LDXR yielded approximately unbiased estimates of relative enrichment/depletion of squared trans-ethnic genetic correlation for the null and causal simulations. S-LDXR was applied to ~93K East Asians and 274K Europeans using GWAS summary statistics for 30 phenotypes, which included diseases and complex traits. They meta-analyzed results across traits and find a depletion of squared trans-ethnic genetic correlation in functionally important regions, which the authors conclude implies more population-specific causal effect sizes. This phenomenon was observed when using annotation based on the top quintile for a background selection statistic, CpG content and SNP-specific Fst. They also applied the S-LDXR method using gene expression annotations for 53 tissues. For each tissue, squared trans-ethnic genetic correlation estimates were estimated and meta-analyzed across the 30 phenotypes for the European and East Asian summary statistics. There was depletion of squared trans-ethnic genetic correlation estimates for all 53 tissues, and the authors conclude that causal disease effect sizes are more population-specific in regions surrounding specifically expressed genes.

This is paper that builds on the work of the Brown et al. paper entitled "Trans-ethnic Genetic-Correlation Estimates from Summary Statistics" [AJHG 99:66-88; 2016] where differences in average genetic correlation for a pair of populations for different classes of SNPs based on functional annotation are assessed relative to genome-wide averages. I find that the S-LDXR results that identifies a deficit in squared trans-ethnic genetic correlation for SNPs in functional regions to be interesting. However, the S-LDXR does not appear to be providing reliable or consistent estimates of deficit/enrichment of squared trans-ethnic genetic correlation in many of the simulation settings. In Figures 1a and 1b, the true casual enrichment/deficit value is outside of the standard error bars for both the continuous and binary annotations. This is problematic and has serious implications for the reliability of the results presented in the real data applications.

Also, additional work is needed to provide sufficient insight into plausible causes of deficit or enrichment of squared trans-ethnic genetic correlation. In the discussion, the authors note that reductions in trans-ethnic genetic correlation as inferred by the S-LDXR could be caused by gene-environment (G   E) interaction and gene-gene (G   G) interaction, for example. However, this was not explored in the simulation studies. How robust is the S-LDXR method? A more thorough investigation of S-LDXR is needed.

I have a few additional comments for the authors.

Comments:

1. It is stated on lines 186-187 on page 7 that the average genetic correlations across 30 traits was around 0.83 for European and East Asians. This seems quite high, particularly in light of previously mentioned Brown et al. [AJHG 99:66-88; 2016] paper where "mean trans-ethnic genetic correlation across all genes was low" for Europeans and East Asians, with an average around 0.32. The authors should provide some insight into this.

2. Is the genome-wide average genetic correlation used in S-LDXR calculated using all SNPs, even those that are not in functional regions, or is it it based on the average genetic correlation across all of the different functional annotations considered? This wasn't clear to me.

3. The S-LDXR provides a statistic measuring enrichment/depletion, but it is not clear to me what the formal statistical test being used to assess deviations from the null hypothesis. How is the test being conducted for continuous value annotation that is split into quintiles as well as for binary annotations? Additional material is needed on this and should appear prominently in the manuscript.

4. Related to comment 3 above, the authors should provide type I error a power estimates (with standard errors) for all of the simulation studies considered.

5. The authors largely focus on depletion of genetic correlation. Under what settings would enrichment be expected? It would be helpful if the authors provide some material and/or a discussion about this.

6. The authors compare European and Asian populations. How would the results change when considering more distant continental populations, such as European vs. African populations or Asian vs. African populations? What impact does the level of divergence of the two populations have on average genetic correlation and the S-LDXR statistic for measuring enrichment/depletion?

7. The authors recommend using alpha= 0.5 as the default shrinkage parameter for the S-LDXR shrinkage estimator based on the simulation studies. Is this shrinkage value expected to be appropriate for other populations, such as, such as European and African populations? In practice, how should this shrinkage value be identified?

8. It would be helpful for the authors to provide insight as to how the S-LDXR statistic behaves when the two populations have the same causal variants but different allele frequencies, as well as when the causal variants are different. It isn't clear to me if the authors considered these settings in the simulation studies.

9. For GWAS, the true "causal" variants may not have been genotyped, so SNPs that may be in LD with the casual variant are used for assessing trans-ethnic genetic correlations instead of the true casual variants. How does using SNPs in LD with the causal variant impact the S-LDXR statistic when there are differential LD patterns in the causal region across populations? It would be helpful for the authors to provide some insight into this setting, which often occurs in GWAS.

Reviewer #3 (Remarks to the Author):

The authors describe a novel method to measure trans-ethnic squared genetic correlation, or a way to quantify the enrichment/depletion of similarity of causal effect sizes between populations. This new method can be useful in identifying annotations that may have increased population-differentiation of causal effect sizes, to hopefully downstream improve prediction and understanding of the underlying biology of traits. They provide simulations and the application of these methods to 30 traits comparing Europeans and Japanese summary statistics.

In general, the manuscript would benefit from an expansion of potential uses for this novel method and interpretations of results when applied across traits. It is unclear as it currently stands why other investigators would adopt this method, especially in a trait-specific manner as the majority of analyses are conducted as a meta-analysis across all traits. This would require expansion in both the Introduction and Discussion sections.

Major Comments

- The manuscript is currently written with much of the methods in the results section. This is understandable, as it is a methods development manuscript, but for this specific audience it should be rewritten with the methods in the results section simplified and the vast majority of this text in the methods section.
- All of the trait-related results are presented as a meta-analysis of all 30 diseases and complex traits. It would be great to have a section discussing trait- or trait group-specific trends. For example, do the two anthropometric traits (height, BMI) show different patterns, given that their heritability is much different? It would be beneficial to expand discussion of these trait-specific trends to inform the reader of possible uses for this method in more trait focused projects.
- A possible implication is cited as reweighting PRS from European population training data based on enrichment/depletion of the squared trans-ethnic genetic correlation. How would this practically be done in respect to directionality of effect weights? Would this just be downweighting SNPs that are depleted (therefore more likely to be different) and upweighting SNPs that are enriched (more likely to transfer)? And subsequently, would this improve performance or trend towards the null in terms of prediction if the larger effect sizes are in differentiated regions? It would be great to expand on this point in the discussion, given it is also mentioned in the introduction.
- The authors note a limitation is that gene expression measurements were conducted in Europeans and may not be applicable to non-European groups. When looking at trans-ethnic correlations and annotation categories, which reference group do you pick for these annotations? These analyses were conducted in the European-derived reference datasets. Would you need to do two analyses, one in European and one in Asian reference sets, and then compare/contrast?
- There is currently not a description of the summary statistics used for trait-specific analyses. For example, were these summary statistics genome-wide on a genotyping array or after imputation to 1000Genomes or another reference panel? Besides MAF cut-offs, were there any other thresholds used to select inclusion? As many PRS use a thresholded approach, how would this affect the weighting if the depletion/enrichment were limited to SNPs above a certain p-value threshold, which would mean larger effect sizes given their common frequencies of MAF>5%?
- Top 5% of simulations with highest standard errors were discarded to assess unbiasedness of estimator. However, the authors note that in the analysis of real traits, these estimates would contribute very little to the meta-analysis across traits. Does this mean this method should only be used across multiple traits and not for a few traits on their own?
- The method compares the similarity versus dissimilarity of effect sizes across different populations. However, it would also be useful to include a discussion of effect size magnitudes, which ties into the heritability and the number of SNPs considered.

Minor Comments

- Figures S4-6: The legends for these figures are confusing. For each of these figures, the legends only refer to panels a and b (S4), c and d (S5), or e (S6). The legends should be expanded to explicitly state the differences between all panels.
- Figure S7: What are the blue and red filled dots symbolizing?

Response to reviewers for NCOMMS-19-37936-T (Shi et al.)

We thank all the reviewers for their helpful comments. Point-by-point responses to all reviewer comments are provided below. We first list some additional changes that we have made to the manuscript, motivated by the reviewer comments but distinct from the changes directly requested by the reviewers. All changes to the manuscript are highlighted in red font.

Additional Changes:

1. We have improved the bias correction of our S-LDXR method for estimating the enrichment/depletion ($\lambda^2(C)$) of stratified squared trans-ethnic genetic correlation ($r_g^2(C)$) of annotation C. Briefly, we analytically derived and corrected for the bias in estimates of $r_g^2(C)$ and $\lambda^2(C)$. In null simulations, our new estimator is more robust in estimating $\lambda^2(C)$ of small annotations (proportion of SNPs < 1%). This is an improvement from our previous jackknife approach to correct for bias in estimating $\lambda^2(C)$, which had limited capacity to correct for bias for small annotations.

We have updated the *Overview of methods* subsection of the *Results* section to briefly mention the analytical bias correction (see page 4-5), and updated the *Methods* section (see page 19) to describe the new estimator.

2. We have updated the interpretation of the depletion of squared trans-ethnic genetic correlation at SNPs surrounding genes specifically expressed in ovary. In detail, a recent study (Li et al. 2018 *Am J Hum Genet*, ref. 40) showed that the *PGR* gene, specifically expressed in ovary, is impacted by recent positive selection. This result is consistent with our hypothesis that stronger gene x environment (G×E) interaction at loci impacted by positive selection induces population-specific causal effect sizes.

We have updated the *Analysis of specifically expressed gene annotations* subsection of the *Results* section (see page 10) to discuss this interpretation.

3. We have now analyzed East Asian and European GWAS summary statistics for schizophrenia (Lam et al. 2019 *Nat Genet*, ref. 98), a psychiatric disorder whose underlying genetic variants are strongly impacted by negative selection (Pardiñas et al. 2018 *Nat Genet*). We observed high genome-wide trans-ethnic genetic correlation (0.95 (s.e. 0.04)). This result suggests that negative selection likely has limited impact on inducing population-specific causal effect sizes, corroborating our hypothesis involving G×E at loci impacted by positive selection.

We have updated the *Analysis of specifically expressed gene annotations* subsection of the *Results* section (see page 10) to discuss the results for schizophrenia.

Reviewer #1 (Remarks to the Author):

In this paper, the authors introduce a new member of their LDSC methodology family, called S-LDXR, to estimate the trans-ethnic genetic correlations stratified by genomic functional annotations using GWAS summary statistics. Building on two previous notable LDSC models: bivariate-LDSC for estimating genetic correlations between traits in one population, and S-LDSC for estimating per-SNP heritability enrichment in functional annotations, this method combines the merit of the two and allows the genetic correlations between different ethnic populations to be variable across functional annotations, taking the population-specific LD into account. The authors apply the method on 30 diseases and complex traits in East Asians (EAS) and Europeans (EUR) with established genomic and tissue-specific annotations, in attempt to understand the biology and evolutionary cause underlying population-specific causal effect sizes.

We thank the reviewer for accurately summarizing our study.

Overall, I find the paper to be quite good and very well written. The methodology is solid with a lot of details and the results are very interesting. The authors conclude that strong GxE interaction at regions undergone positive selection is the most likely mechanism to give rise to population-specific causal effect sizes. While it is plausible, I have following concerns/questions to be addressed before I can fully agree. I hope the authors will find my comments useful.

We thank the reviewer for suggesting that our paper is very interesting and very well written. Reviewer concerns/questions are addressed below.

Major:

1. The authors claim that the squared trans-ethnic genetic correlation (rg^2) is defined at causal effect sizes, so any depletion in lambda^2(C) = rg^2(C)/rg^2 observed for a functional category (C) must be due to the differences in causal effect sizes between the two ethnic populations. It is a bit hard for me to really believe that their rg^2 captures all causal effects, given that only HapMap3 common SNPs (MAF>5%) are included in the analysis and the various limitations in genotyping and imputation process. So, it is still a question for me that if a depletion in lambda^2(C) is due to the differences in causal effect sizes or due to the differences in LD and allele frequencies of the unobserved causal variants. This problem may become bigger when the causal variants with large effect sizes tend to be rarer in one population than in the other population due to differential actions of negative selection. Additionally, unlike S-LDSC, the S-LDXR does not include MAF bins as annotations, and the previous work has shown that the heritability estimate may be biased without stratifying SNPs by MAF, especially when the causal variants are enriched in low MAF bin. Thus, I suggest to investigate the unbiasness of lambda^2(C) estimate, as compared to that at the causal effects, by simulations that accounts for the properties of rare causal variants. For example, they can consider a model of negative selection where causal effect sizes are inversely related to MAF (with the relationship they found in Schoech et al 2019) and vary the proportion of causal variants being rare across functional categories.

The reviewer has raised several related questions: (a) Do the results pertain to HapMap3 SNPs only, given that only HapMap3 SNPs are analyzed; (b) Does S-LDXR attain robust results for annotation-dependent MAF-dependent architectures; (c) Should S-LDXR include MAF bins as annotations. We address each of these questions in turn.

(a) Do the results pertain to HapMap3 SNPs only, given that only HapMap3 SNPs are analyzed.

S-LDXR analyzes summary statistics from HapMap3 SNPs with MAF > 5% in both populations (*regression SNPs*) together with reference panel LD from SNPs present in either population in 1000 Genomes (*reference SNPs*) to estimate enrichment/depletion (defined as a function of causal effect sizes) for all SNPs with MAF > 5% in both populations (*heritability SNPs*), accounting for tagging effects (analogous to S-LDSC; Finucane et al. 2015 *Nat Genet*, ref. 21). Thus, the results pertain to causal effects of all SNPs with MAF > 5% in both populations (*heritability SNPs*). We recognize that it is our responsibility to verify that not just the definition but also the actual estimates achieve this result. Thus, we simulated traits using SNPs present in either population in 1000 Genomes (including non-HapMap3 SNPs) as causal SNPs in our simulations, including simulations with annotation-dependent MAF-dependent architectures.

We have updated the *Introduction* section (see page 3), *Overview of methods* subsection of the *Results* section (see pages 4-5) and *Methods* section (see pages 18-19) to clarify this point. (Also see response to Reviewer 1 Comment 4 and response to Reviewer 2 Comment 9).

(b) Does S-LDXR attain robust results for annotation-dependent MAF-dependent architectures.

Motivated by the reviewer's question, we performed simulations with annotation-dependent MAF-dependent architectures, defined as architectures in which the level of MAF-dependence is annotation-dependent. We determined that S-LDXR yielded nearly unbiased estimates of enrichment/depletion of stratified squared trans-ethnic genetic correlation ($\lambda^2(C)$) for binary functional annotations (Figure S8, Table S10), unbiased estimates for quintiles of most continuous-valued annotations (Figure S9, Table S11), and slightly biased estimates in the top and bottom quintile of the average level of LD annotation and the recombination rate annotation (Figure S9, Table S11; the bias is small compared to the enrichment/depletion we observed in analyses of real traits, and we conjecture that it is because of imperfect tagging of rare causal variants due to small sample size the reference panel).

We have updated the *Simulations* subsection of the *Results* section (see pages 5-6) and the *Methods* section (see page 23) to describe these new simulations.

(c) Should S-LDXR include MAF bins as annotations.

We decided not to include MAF bin annotations in the baseline-LD-X model used by S-LDXR, for two reasons. First, we estimated $\lambda^2(C)$ in quintiles of MAF (maximum across 2 populations) and did not observe significant enrichment/depletion of squared trans-ethnic genetic correlation (Table S16), suggesting that squared trans-ethnic genetic correlation does not depend on MAF. Second, in simulations with annotation-dependent MAF-dependent

architectures, we determined that S-LDXR with MAF bins attained results that were very similar to S-LDXR without MAF bins (Figure S8, S9, Table S10, S11).

We have updated the *Simulations* subsection of the *Results* section (see pages 6-7) and the *Analysis of baseline-LD-X model annotations across 31 diseases and complex traits* subsection of the Results section (see page 9) to describe these results.

2. In their simulation study for assessing the estimation of rg and lambda^2(C), they vary the true rg from 0.20 to 0.96, which is a bit odd. How about rg = 0 and rg = 1? The estimation performance at the extreme values of rg is important as there is a high interest especially in testing for rg = 1. Moreover, the EAS and EUR populations have roughly equal GWAS sample sizes in the simulation, which are actually very different in practice. The authors should simulate different sample sizes with a ratio similar to that in real data.

The reviewer has made two requests: (a) simulations with a ratio of EAS vs. EUR sample size similar to that in real data, and (b) simulations at extreme values of r_g. We address each of these requests in turn.

(a) simulations with a ratio of EAS vs. EUR sample size similar to that in real data.

We have modified all simulations in our paper so that the East Asian sample sizes is half of the European sample size, analogous to real data.

We have updated the *Simulations* subsection of the *Results* section (see page 5), Figure 1, all simulation Supplementary Tables/Supplementary Figures, and the *Methods* section (see page 22) accordingly.

(b) simulations at extreme values of r_g.

Simulations assessing estimation of r_g: we performed simulations in which r_g was set to 0 or 1. We determine that S-LDXR yielded accurate estimate of r_g in these simulations (Figure S3, Table S3).

Simulations assessing estimation of $\lambda^2(C)$: our previous simulations considered r_g=0.8 only. We elected not to vary r_g in these simulations, as r_g's of most of diseases and complex traits analyzed in this work are close to 0.8. Instead, we performed simulations in which 20% of causal variants were specific to each population (also see response to Reviewer 2 Comment 7). S-LDXR yielded nearly unbiased estimates of $\lambda^2(C)$ in these simulations, just as in our main simulations (see Figure S11, Table S12).

We have updated the *Simulations* subsection of the *Results* section (see page 5, page 7) and the *Methods* section (see page 23) to describe these new simulations.

3. The power to detect enrichment or depletion in a binary annotation seems to be quite variable across different functional annotations (Figure 1b). While the reduction of power does not appear to be related to the annotation size, I wonder what factors affect the power, which

could be important to understand any confounding factors in the results of real data analysis. For example, is the power related to the average LD score in the annotation?

We thank the reviewer for pointing out the lack of discussion of potential factors impacting the power to detect enrichment/depletion of stratified squared trans-ethnic genetic correlation ($\lambda^2(C)$).

The standard error of $\lambda^2(C)$ for a binary annotation primarily depends on the total heritability of SNPs in the annotation (sum of per-SNP variances of standardized causal effect sizes), which appears as the denominator ($h_{g1}^2(C)h_{g2}^2(C)$) in the estimation of stratified squared trans-ethnic genetic correlation ($r_g^2(C)$); if this denominator is small, estimation of $r_g^2(C)$ becomes noisy.

The standard error of $\lambda^2(C)$ for a binary annotation indirectly depends on the size of the annotation, because larger annotations tend to have larger total heritability. However, estimates of $\lambda^2(C)$ for a large annotation may have large standard error if the annotation is depleted for heritability.

We have updated the *Overview of methods* subsection of the *Results* section to briefly mention factors impacting the power of S-LDXR (see page 4-5), citing a more detailed discussion of this in the *Methods* section (page 19).

4. They find that regions with strong depletion in lambda^2(C) are those with prior evidence of positive selection (coding, conserved, regulatory regions), and GxE interaction is a plausible mechanism for the differences in effect sizes across environments, therefore conclude that GxE interaction together with the influence of positive selection have resulted in causal effect sizes to be different across populations. However, these functional regions with lambda^2(C) depletion have also been found to have signatures of negative selection (especially conserved region). Related to my comment #1, the effect sizes at the common SNPs may appear to be different if the rare (or low-frequency) causal variant effect sizes are large and the LD between the rare variants and common SNPs are different between populations. In addition, the depletions of lambda^2(C) in the top quintile of background selection statistic and CpG content as well as the bottom quintile of nucleotide diversity and recombination rate (Figure 2b) seem to be more relevant to the model of negative selection. It seems the hypothesis of negative selection even without GxE can explain these results as well. How to reconcile these two hypotheses?

The reviewer has raised two related questions: (a) are $\lambda^2(C)$ results for common SNPs impacted by differential tagging of rare variants across populations, and (b) can negative selection (instead of positive selection) explain our findings. We address each of these questions in turn.

(a) are $\lambda^2(C)$ results for common SNPs impacted by differential tagging of rare variants across populations.

As noted in part (a) of our response to Reviewer 1 Comment 1, S-LDXR analyzes summary statistics from HapMap3 SNPs with MAF > 5% in both populations (*regression SNPs*) together with reference panel LD from SNPs present in either population in 1000 Genomes (*reference SNPs*) to estimate enrichment/depletion (defined as a function of causal effect sizes) for all SNPs with MAF > 5% in both populations (*heritability SNPs*), accounting for tagging effects (analogous to S-LDSC; Finucane et al. 2015 *Nat Genet*, ref. 21). Thus, the results pertain to causal effects of all SNPs with MAF > 5% in both populations (*heritability SNPs*). Our simulations, including simulations with annotation-dependent MAF-dependent architectures, verify that not just the definition but also the actual estimates achieve this result. Thus, $\lambda^2(C)$ estimates (which pertain to causal effects of all SNPs with MAF > 5% in both populations) are not impacted by differential tagging of rare variants across populations.

We have updated the *Introduction* section (see page 3), *Overview of methods* subsection of the *Results* section (see pages 4-5) and *Methods* section (see pages 18-19) to clarify this point.

(b) can negative selection (instead of positive selection) explain our findings.

The reviewer is correct that annotations such as background selection statistic can reflect the action of both positive selection and negative selection (McVicker et al. 2009 *PLoS Genet*, ref. 30). Nonetheless, we believe that our overall set of results is better explained by positive selection (e.g. stronger gene-environment interaction at loci impacted by positive selection) than by negative selection, for the following reasons:

(i) We observed substantial depletion of squared trans-ethnic genetic correlation for SNPs near skin ($\lambda^2(C) = 0.83\ (s.e. 0.02)$ for Skin Sun Exposed (Lower Leg)) and immune-related genes ($\lambda^2(C) = 0.85\ (s.e. 0.02)$ for Spleen), which are strongly impacted by recent positive selection, but not for SNPs near brain genes (($\lambda^2(C) = 0.98\ (s.e. 0.02)$ for Brain Nucleus Accumbens (Basal Ganglia)) (see Figure 4, Table S20).

(ii) We observed a lack of variation in enrichment/depletion of squared trans-ethnic genetic correlation across genes in different deciles of probability of loss-of-function intolerance (see Figure S24, Table S23).

(iii) As noted in Additional Change 2, we have updated the interpretation of the depletion of squared trans-ethnic genetic correlation at SNPs surrounding genes specifically expressed in ovary. Specifically, a recent study (Li et al. 2018 *Am J Hum Genet*, ref. 40) showed that the *PGR* gene, specifically expressed in ovary, is impacted by recent positive selection. This result is consistent with our hypothesis that stronger gene x environment (G×E) interaction at loci impacted by positive selection induces population-specific causal effect sizes. We have updated the *Analysis of specifically expressed gene annotations* subsection of the *Results* section (see page 10) to discuss this interpretation.

(iv) As noted in Additional Change 3, we have now analyzed East Asian and European GWAS summary statistics for schizophrenia (Lam et al. 2019 *Nat Genet*, ref. 98), a psychiatric disorder

whose underlying genetic variants are strongly impacted by negative selection (Pardiñas et al. 2018 *Nat Genet*). We found no significant deviation of genome-wide trans-ethnic genetic correlation from 1 (r_g=0.95 (s.e. 0.04)). This result suggests that negative selection likely has limited impact on inducing population-specific causal effect sizes, corroborating our hypothesis involving G×E at loci impacted by positive selection. We have updated the *Analysis of specifically expressed gene annotations* subsection of the *Results* section (see page 10) to discuss the results for schizophrenia.

(v) (Also see response to Reviewer 1 Comment 7) We performed a new analysis of a binary annotation reflecting the action of positive selection based on the iHS score (Voight et al. 2006 *PLoS Biol*, ref. 43; Johnson & Voight 2018 *Nat Ecol Evol*, ref. 44). We observed strong depletion of squared trans-ethnic genetic correlation ($\lambda^2(C)$=0.88 (s.e. 0.03)). This annotation is positively correlated with the background selection statistic annotation (R=0.08), confirming that SNPs with high background selection statistic can indeed reflect the action of positive selection. We have updated the *Analysis of specifically expressed gene annotations* subsection of the *Results* section (see page 10) and the Methods section (see page 26) to describe the iHS analysis.

We have updated the discussion of positive selection vs. negative selection in the *Discussion* section (see page 13) to include all of these reasons for favoring the positive selection hypothesis, while noting that we cannot formally exclude explanations involving negative selection.

5. In Figure 2b, most annotations show a pattern that the enrichment or depletion of genetic correlation (lambda^2(C)) tends to be negatively related to that of heritability (h^2(g)) across quintiles. According to the argument in the paper, this could be the result of a combination of stronger GxE interaction and positive selection at functionally important regions (therefore higher per-SNP heritability). Then I find it is hard to interpret the clearly opposite patterns observed in the average LLD and GERP (NS), i.e. lambda^2(C) is positively related to h^2(C). The authors briefly mention it in the results but I find the explanation is a bit confusing.

We agree with the reviewer that more discussion of the relationship between enrichment of heritability and enrichment/depletion of squared trans-ethnic genetic correlation is warranted, given the negative relationship for most annotations (higher heritability, and depletion of squared trans-ethnic genetic correlation) but positive relationship for the top quintile of average LLD and the bottom quintile of GERP (NS) (lower heritability, but depletion of squared trans-ethnic genetic correlation). We have added a sentence in the *Discussion* section (see page 12) discussing this relationship.

We believe that the fundamental cause of depletion of trans-ethnic genetic correlation is gene-environment interaction (G×E), not heritability enrichment, although there is indeed a general trend that annotations enriched for heritability show depletion of squared trans-ethnic genetic correlation. Therefore, an annotation enriched for SNPs exhibiting G×E may show depletion of squared trans-ethnic genetic correlation, even if the annotation is depleted of heritability.

We believe that the depletion of squared trans-ethnic genetic correlation in the top quintile of the average LLD annotation is due to stronger G×E. Additionally, the top quintile of the average LLD annotation is likely to be impacted by positive selection, as recent positive selection could increase the frequency of the selected haplotypes, which are typically longer due to their younger age, leading to higher LD (Sabeti et al. 2002 *Nature*, Voight et al. 2006 *PLoS Biol*). The depletion of squared trans-ethnic genetic correlation in the bottom quintile of the GERP NS annotation can be partially explained by the moderate negative correlation between the GERP NS annotation and the average LLD annotation (R=-0.13).

Finally, the depletion of $\lambda^2(C)$ in these quintiles could also be explained by their correlation with the background selection statistic annotation (see Figure S2 and Table S1) – the S-LDXR method cannot disentangle $\lambda^2(C)$ among correlated annotations, as genetic correlation is a quotient of trans-ethnic genetic covariance and square root of product of heritabilities. We discuss this limitation of S-LDXR in the limitations paragraph of the *Discussion* section (see page 15).

6. In the Supplementary Note, they have a section describing a two-population Eyre-Walker model and find that a result similar to the real data analysis can be observed only when the fitness effects for deleterious SNPs differ in both mean and variance across populations. I find this to be interesting. If this is true, we can expect that the relationship between effect sizes and MAF will be different in the two populations. Given that there are methods available to estimate such a relationship using GWAS summary data (Gazal et al 2018 NG; Speed et al 2019 bioRxiv; Zeng et al 2019 bioRxiv), it would be interesting to test that in their data.

We thank Reviewer 1 for their interest in our two-population Eyre-Walker model. However, we respectfully disagree with the conclusion that we expect the relationship between effect size and MAF to be different in the two populations. Under our model, fitness effects in the two populations have the same mean, but higher variance at SNPs with large fitness effect (i.e. strongly deleterious SNPs) and lower variance at SNPs with small fitness effect (i.e. weakly deleterious SNPs). Since both populations have the same mean fitness effect, we expect the relationship between effect size and MAF to be the same in the two populations for strongly deleterious SNPs, and we also expect the relationship between effect size and MAF to be the same in the two populations for weakly deleterious SNPs. As we cannot think of any reason to expect the relationship between effect size and MAF to be different in the two populations, we elected not to test this in real data, which would be a very substantial undertaking, likely producing a new manuscript in itself.

We caution that our proposed two-population Eyre-Walker model is a "proof-of-concept" explanation for the depletion of squared trans-ethnic genetic correlation in functionally important regions, demonstrating that population-specific causal disease effect sizes can *in principle* be explained by population-specific fitness effects. This model should not be viewed as a model that is confirmed by results on real traits.

We have updated the Supplementary Note (see page 11) to clarify these points.

7. Besides SNP-specific Fst, have they looked at other per-SNP positive selection annotations, especially those for detecting recent selection?

We thank the reviewer for this suggestion. We performed a new analysis of a binary annotation reflecting the action of positive selection based on the iHS score (Voight et al. 2006 *PLoS Biol*, ref. 43; Johnson & Voight 2018 *Nat Ecol Evol*, ref. 44). We observed strong depletion of squared trans-ethnic genetic correlation ($\lambda^2(C)$=0.88 (s.e. 0.03)). This result strengthens our conclusion that gene-environment interaction at loci impacted by positive selection likely contributes to population-specific causal disease effect sizes. (Also see part (v) of part (b) of Response to Reviewer 1 Comment 5.)

We have updated the *Analysis of specifically expressed gene annotations* subsection of the *Results* section (see page 10) and the *Methods* section (see page 26) to describe the iHS analysis.

Minor:

Line 90: It is not clear what C and C' refer to and why C' is needed here.

We thank Reviewer 1 for pointing out the lack of clarity on the meaning of C and C'.

Here, the C in the outer summation is the binary annotation under consideration – the outer summation sums per-SNP trans-ethnic genetic covariance of each SNP j in annotation C. And the C' in the inner summation is an index of annotation – the inner summation sums the contribution of each annotation across the set of all baseline-LD-X model annotations to obtain the per-SNP trans-ethnic genetic covariance of SNP j.

We have updated the *Overview of methods* subsection in the *Results* section (page 4) and *Methods* section (page 17) to clarify this point.

Why they estimate lambda^2(C) for binary annotations only or quintiles of continuous-valued annotations? Is there a technical difficulty to estimate that for a continuous annotation as a whole?

Defining stratified squared trans-ethnic genetic correlation ($r_g^2(C)$) and its enrichment ($\lambda^2(C)$) is challenging for continuous-valued annotations, as squared correlation is a non-linear term involving a quotient of squared covariance and a product of variances. We elected to instead estimate $\lambda^2(C)$ for quintiles of continuous-valued annotations (analogous to our previous work; Gazal et al. 2017 *Nat Genet*, ref. 22) as an intuitive substitute.

We have updated the *Methods* section (see page 18) to clarify this point.

Line 419: As they define beta as per-allele effect size, the sum of per-SNP effect variance is not per-SNP heritability unless multiply by heterozygosity 2pq.

Reviewer 1 is correct that the sum of per-allele effect variances is not equal to heritability on the standardized scale. We have updated the *Methods* section (see page 17), using the term "allelic-scale heritability", to clarify this point.

Figure S4 legend: panel # are incorrectly cited. Also, in "Shrinkage level, α, was set to 0.75 in a", is the number 0.75 correct? I thought it would only make sense if alpha is less than 0.5. Otherwise, the pattern does not seem to be coherent with Figure 1a, where alpha is 0.5 by default.

We thank Reviewer 1 for pointing out the inaccurate legend for Figure S4.

Each panel in each Figure S4 corresponds to simulation results obtained with different shrinkage parameters (α=0.0, 0.25, 0.75, and 1.0 in panel a, b, c, and d, respectively). We did not include the result for α=0.5 in Figure S4, as this result is shown in Figure 1a.

We have updated the legend of Figure S4 accordingly.

Figure S5 legend: the difference between a/b and c/d is the later includes a flanking window of 500bp for each annotation?

We thank Reviewer 1 for pointing out the inaccurate legend for Figure S5.

Each panel in Figure S5 corresponds to simulation results for binary functional annotations obtained with different shrinkage parameters (α=0.0, 0.25, 0.75, and 1.0 in panel a, b, c, and d, respectively). We did not include the result for α=0.5 in Figure S5, as this result is shown in Figure 1b. We have updated the legend of Figure S5 accordingly.

Reviewer 3 also pointed out inaccurate legend for Figure S6 (see Reviewer 3 minor comment 1). In Figure S6, each panel corresponds to simulation results for 500bp-extended binary functional annotations obtained with different shrinkage parameters (α=0.0, 0.25, 0.5, 0.75, and 1.0 in panel a, b, c, d, and e, respectively). We have updated the legend of Figure S6 accordingly.

Line 450: tau_1C and tau_2C.

We thank Reviewer 1 for pointing out this typo. We have fixed this typo in our updated manuscript (see page 18).

Reviewer #2 (Remarks to the Author):

The authors consider the problem of estimating genome-wide trans-ethnic genetic correlations. A new method named S-LDXR is proposed to measure trans-ethnic correlations for SNPs in different functional annotation categories. The S-LDXR method incorporates summary statistics from genome-wide association studies (GWAS) and linkage disequilibrium (LD) measures from reference panels The goal of S-LDXR is to identify functional annotation categories that have enrichment or depletion of squared trans-ethnic genetic correlation, where enrichment or depletion is calculated relative to genome-wide average genetic correlation. S-LDXR was evaluated in simulations studies using genotypes that were simulated based on haplotypes from East Asian and European populations. The authors find that estimated genome-wide average correlations for the two simulated populations were approximately unbiased for the simulated true values across different functional categories, and that S-LDXR yielded approximately unbiased estimates of relative enrichment/depletion of squared trans-ethnic genetic correlation for the null and causal simulations. S-LDXR was applied to ~93K East Asians and 274K Europeans using GWAS summary statistics for 30 phenotypes, which included diseases and complex traits. They meta-analyzed results across traits and find a depletion of squared trans-ethnic genetic correlation in functionally important regions, which the authors conclude implies more population-specific causal effect sizes. This phenomenon was observed when using annotation based on the top quintile for a background selection statistic, CpG content and SNP-specific Fst. They also applied the S-LDXR method using gene expression annotations for 53 tissues. For each tissue, squared trans-ethnic genetic correlation estimates were estimated and meta-analyzed across the 30 phenotypes for the European and East Asian summary statistics. There was depletion of squared trans-ethnic genetic correlation estimates for all 53 tissues, and the authors conclude that causal disease effect sizes are more population-specific in regions surrounding specifically expressed genes.

We thank Reviewer 2 for accurately summarizing our study. We would like to clarify two minor points (see text in yellow highlight above).

(a) The S-LDXR method yields unbiased estimates of enrichment/depletion of stratified squared trans-ethnic genetic correlation ($\lambda^2(C)$) in *null* simulations. However, S-LDXR is conservatively biased towards the null ($\lambda^2(C) = 1$) in *causal* simulations (particularly for binary annotations of small size), due to our shrinkage estimator. We have updated the *Simulations* subsection of the *Results* section (see pages 5-6) to clarify this point.

(b) For specifically expressed gene annotations, we did not observe significant depletion of squared trans-ethnic genetic correlation at SNPs surrounding genes specifically expressed in brain tissues (see Figure 4), which are impacted by negative selection. (The strongest depletion of squared trans-ethnic genetic correlation was observed for genes specifically expressed in skin and immune tissues (see Figure 4), which are impacted by positive selection). We have updated the *Analysis of specifically expressed gene annotations* subsection of the *Results* section (see page 10) to clarify this point.

This is paper that builds on the work of the Brown et al. paper entitled "Trans-ethnic Genetic-Correlation Estimates from Summary Statistics" [AJHG 99:66-88; 2016] where differences in average genetic correlation for a pair of populations for different classes of SNPs based on functional annotation are assessed relative to genome-wide averages. I find that the S-LDXR results that identifies a deficit in squared trans-ethnic genetic correlation for SNPs in functional regions to be interesting. However, the S-LDXR does not appear to be providing reliable or consistent estimates of deficit/enrichment of squared trans-ethnic genetic correlation in many of the simulation settings. In Figures 1a and 1b, the true casual enrichment/deficit value is outside of the standard error bars for both the continuous and binary annotations. This is problematic and has serious implications for the reliability of the results presented in the real data applications.

We thank the reviewer for suggesting that our results on real traits are interesting. The reviewer concerns about the robustness of S-LDXR in simulations are addressed below.

We emphasize that the S-LDXR method is approximately unbiased in *null* simulations, in which the true enrichment/depletion of stratified squared genetic correlation ($\lambda^2(C)$) is equal to 1 (Figure 1a and Figure 1b). We have updated the *Simulations* subsection of the *Results* section (see pages 5-6) to clarify this point. (Also see part (a) of response to Reviewer 2 Introductory comments, paragraph 1).

The reviewer is correct that S-LDXR is biased in *causal* simulations. Specifically, S-LDXR is conservatively biased towards the null ($\lambda^2(C)$=1) in causal simulations (particularly for binary annotations of small size). The bias in causal simulations is due to the shrinkage estimator that we applied to reduce variance in the estimated $\lambda^2(C)$ (see *Overview of methods* subsection of *Results* section (page 4-5) and *Methods* section (page 20)). The shrinkage estimator shrinks the estimates of $\lambda^2(C)$ towards the null ($\lambda^2(C) = 1$). Thus, we believe that for real traits, the true enrichment/depletion of stratified trans-ethnic genetic correlation is stronger than our estimates. We have updated the *Simulations* subsection of the *Results* section (see page 6) to clarify this point. (Also see part (a) of response to Reviewer 2 Introductory comments, paragraph 1).

Also, additional work is needed to provide sufficient insight into plausible causes of deficit or enrichment of squared trans-ethnic genetic correlation. In the discussion, the authors note that reductions in trans-ethnic genetic correlation as inferred by the S-LDXR could be caused by gene-environment (G×E) interaction and gene-gene (G×G) interaction, for example. However, this was not explored in the simulation studies. How robust is the S-LDXR method? A more thorough investigation of S-LDXR is needed.

The reviewer has raised two separate questions: (a) Could simulation studies shed light on the hypothesized role of G×E interaction, and (b) How robust is the S-LDXR method. We address each of these questions in turn.

(a) Could simulation studies shed light on the hypothesized role of G×E interaction.

We hypothesize that G×E is likely the primary cause of depletion of trans-ethnic correlation. If individual-level measurements of environmental factors were available, one could explicitly model and incorporate environmental factors. However, this is beyond the focus and scope of this manuscript, as environmental variables are typically not publicly available (more generally, individual-level data is not publicly available for the East Asian summary statistic data sets that we analyzed). We agree that this is an interesting direction for future research. We note that although we do not explicitly simulate G×E, G×E would induce population-specific causal effect sizes, which we do explicitly simulate in our simulations. We note that we hypothesize a greater role for G×E (supported by e.g. Robinson et al. 2017 *Nat Genet*, ref. 51) than G×G or dominance variation, which have been reported in recent studies to explain little heritability (Hill et al. 2008 *PLoS Genet*, ref. 52; Maki-Tanila and Hill 2014 *Genetics*, ref. 53; Zhu et al. 2015 *Am J Hum Genet*, ref. 54). We have updated the *Discussion* section (see page 14) to discuss these points.

(b) How robust is the S-LDXR method.

The S-LDXR method is approximately unbiased in null simulations, and conservatively biased towards the null in causal simulations. See part (b) of response to Reviewer 1 Comment 1, part (b) of response to Reviewer 1 comment 2, response to Reviewer 2 Introductory comments paragraph 1, and response to Reviewer 2 Introductory comments paragraph 2.

I have a few additional comments for the authors.

Comments:

1. It is stated on lines 186-187 on page 7 that the average genetic correlations across 30 traits was around 0.83 for European and East Asians. This seems quite high, particularly in light of previously mentioned Brown et al. [AJHG 99:66-88; 2016] paper where "mean trans-ethnic genetic correlation across all genes was low" for Europeans and East Asians, with an average around 0.32. The authors should provide some insight into this.

The reviewer is correct that our average trans-ethnic genetic correlation of 0.83 for 30 *complex traits* is higher than the average (cis) trans-ethnic genetic correlation of 0.32 for *gene expression traits* in Brown et al. 2016 *Am J Hum Genet* (Popcorn method, ref. 2). We believe that our average estimate of 0.83—which is still substantially lower than 1—is accurate, because (a) gene expression traits are expected to have a very different genetic architecture than complex traits, (b) Brown et al. reported that their average estimate of 0.32 increases to 0.77 when restricting their analysis to gene expression traits with (cis) heritability greater than 0.2 in both populations, (c) Martin et al. 2019 *Nat Genet* (ref. 6) reported an average trans-ethnic genetic correlation of 0.88 for 22 complex traits, which is similar to our average estimate of 0.83 for 30 complex traits, and (d) our S-LDXR method and the Popcorn method of Brown et

al. produced similar average estimates of trans-ethnic genetic correlation for the complex traits that we analyzed (Figure S13). We have updated the *Analysis of baseline-LD-X model annotations across 31 diseases and complex traits* subsection of the *Results* section to clarify these points (see page 7).

We note that our average estimate of 0.83 across 30 complex traits has changed to an average estimate of 0.85 across 31 complex traits, due to the addition of schizophrenia (see Additional change 3 above).

2. Is the genome-wide average genetic correlation used in S-LDXR calculated using all SNPs, even those that are not in functional regions, or is it it based on the average genetic correlation across all of the different functional annotations considered? This wasn't clear to me.

Our estimates of genome-wide trans-ethnic genetic correlation pertain to causal effects of all SNPs with minor allele frequency (MAF) greater than 5% in both populations (*heritability SNPs*), regardless of whether the SNPs belong to a functional annotation or not. We have updated *Methods* section (page 19) to clarify this point. (Also see part (a) of response to Reviewer 1 Comment 1, part (a) of response to Reviewer 1 Comment 4, response to Reviewer 2 Comment 9, and corresponding updates to the *Introduction* section (see page 3), *Overview of methods* subsection of the *Results* section (see pages 4-5) and *Methods* section (see pages 18-19).)

3. The S-LDXR provides a statistic measuring enrichment/depletion, but it is not clear to me what the formal statistical test being used to assess deviations from the null hypothesis. How is the test being conducted for continuous value annotation that is split into quintiles as well as for binary annotations? Additional material is needed on this and should appear prominently in the manuscript.

When evaluating enrichment/depletion of squared trans-ethnic genetic correlation ($\lambda^2(C)$), the null hypothesis is that there is no enrichment/depletion of squared trans-ethnic genetic correlation, i.e. that $\lambda^2(C)$ is equal to 1.

To test whether stratified squared trans-ethnic genetic correlation is enriched/depleted for an annotation for a trait, we test whether the estimate $\hat{\lambda}^2(C)$ for that annotation is different from 1. This is equivalent to testing $\widehat{\mathrm{D}}^2(\mathrm{C}) = \hat{\rho}_g^2(\mathrm{C}) - \hat{r}_g^2\hat{h}_{g1}^2(\mathrm{C})h_{g2}^2(\mathrm{C})$, where $\hat{r}_g^2$ is the genome-wide squared trans-ethnic genetic correlation. We compute a t-statistic as $\frac{\widehat{\mathrm{D}}^2(\mathrm{C})}{s.e.\left(\widehat{\mathrm{D}}^2(\mathrm{C})\right)}$, and a two-tailed p-value from the t distribution with degree of freedom set to B-1, where B is the number of blocks used in the block-jackknife procedure.

To test whether meta-analyzed squared trans-ethnic genetic correlation is enriched/depleted for an annotation, we compute a t-statistic as $\frac{\hat{\lambda}^2(\mathrm{C})-1}{s.e.\left(\hat{\lambda}^2(\mathrm{C})\right)}$, and a two-tailed p-value from the normal distribution.

The procedure to test for enrichment/depletion in binary functional annotations and in quintiles of continuous-valued annotations are the same. For a binary annotation, C denotes the set of SNPs that belong to the binary annotation. For a quintile of continuous-valued annotation, C denotes the set of SNPs having annotation value in that quintile.

We have updated the *Overview of methods* subsection of the *Results* section (see pages 4-5) and the *Methods* section (see page 21 (new *Significance Testing* subsection) and page 24) to clarify these points. In light of the Reviewer 3 request to reduce the length of the *Overview of methods* subsection of the *Results* section (Reviewer 3 Major comment 1), our updates to *Overview of methods* were necessarily limited to a brief mention of significance testing, citing further details in the *Methods* section, even though Reviewer 2 requested that this material should appear prominently in the manuscript.

4. Related to comment 3 above, the authors should provide type I error a power estimates (with standard errors) for all of the simulation studies considered.

We thank the reviewer for this suggestion. We now include type I error rate for null simulations and power estimates for causal simulations (see Figures S7, S11, Tables S4-S12, cited in the *Simulations* subsection of the *Results* section; see pages 6-7). (Also see response to Reviewer 1 Comment 3 regarding factors impacting power to detect enrichment/depletion.)

5. The authors largely focus on depletion of genetic correlation. Under what settings would enrichment be expected? It would be helpful if the authors provide some material and/or a discussion about this.

Since we hypothesize that *depletion* of trans-ethnic genetic correlation is primarily due to stronger gene-environment interactions in functionally important regions, we expect trans-ethnic genetic correlation to be *enriched* in less functionally important regions, where gene-environment interaction is weaker. Indeed, trans-ethnic genetic correlation is *enriched* for SNPs in the bottom quintile of background selection statistic, which are less functionally important. We primarily focus on *depletion* of trans-ethnic genetic correlation, as our study aims to understand why trans-ethnic genetic correlation of many diseases and complex traits is less than 1.

We have updated the *Discussion* section (page 12) to include a discussion of scenarios where trans-ethnic genetic correlation is expected to be *enriched*.

6. The authors compare European and Asian populations. How would the results change when considering more distant continental populations, such as European vs. African populations or Asian vs. African populations? What impact does the level of divergence of the two populations have on average genetic correlation and the S-LDXR statistic for measuring enrichment/depletion?

Given our hypothesis that imperfect genome-wide trans-ethnic genetic correlation (r_g) is primarily driven by gene-environment interaction, we expect the r_g between African and East Asian populations and the r_g between African and European populations to be similar, if the level of gene-environment interaction (G×E) is similar across these pairs of populations. However, if G×E is stronger in a particular pair of populations, then we expect r_g to be lower.

Accordingly, we expect enrichment/depletion of squared trans-ethnic genetic correlation ($\lambda^2(C)$) to be similar for two pairs of populations if r_g is similar for the two pairs of populations. However, if r_g is lower for a particular pair of populations, then we expect to see higher variance in $\lambda^2(C)$ across annotations, i.e. stronger enrichments/depletions. (Conversely, for two very similar populations with very similar E, we expect both r_g and $\lambda^2(C)$ to be close to 1.)

Unfortunately, we are currently unable to include an analysis of European vs. African or East Asian vs. African populations, both due to the limited number of very large GWAS in African populations (and because most large African-ancestry data sets involve admixed individuals, which introduces additional technical challenges that our methods do not currently address).

We have updated the *Discussion* section (see page 14) to clarify these points.

7. The authors recommend using alpha= 0.5 as the default shrinkage parameter for the S-LDXR shrinkage estimator based on the simulation studies. Is this shrinkage value expected to be appropriate for other populations, such as, such as European and African populations? In practice, how should this shrinkage value be identified?

The reviewer makes a good point that the optimal choice of α may be specific to the pair of populations analyzed. In our simulations, we found that a shrinkage of 0.5 yields conservative estimates of $\lambda^2(C)$ across a wide range of values of polygenicity and power (see Figure 1, Figures S4-S6, Tables S4-S9). Thus, it is reasonable to hypothesize that setting α to 0.5 would also result in conservative estimates for other pairs of populations. However, we recommend that one should ideally perform simulations on the pair of populations being analyzed to select the optimal value of α.

We have updated the *Discussion* section to clarify these points (see page 14).

8. It would be helpful for the authors to provide insight as to how the S-LDXR statistic behaves when the two populations have the same causal variants but different allele frequencies, as well as when the causal variants are different. It isn't clear to me if the authors considered these settings in the simulation studies.

The reviewer has asked about (a) simulations with same causal variants but different allele frequencies across populations, and (b) simulations with different causal variants across populations. We address each of these questions in turn.

(a) simulations with same causal variants but different allele frequencies across populations.

Our current simulation framework involves same causal variants but different allele frequencies across populations. This is because we simulated genotypes (using HAPGEN2; Su et al. 2011 *Bioinformatics*, ref. 25) using real East Asian and European haplotypes, preserving population-specific MAF and LD patterns. We have updated the *Simulations* subsection of the *Results* section (see page 5) and the *Methods* section (page 22) to clarify this point.

(b) simulations with different causal variants across populations.

Motivated by the reviewer's suggestion, we performed additional null simulations in which 80% of causal variants (in either population) are shared across the two populations (with the same per-allele causal effect size), and the remaining causal variants are causal in one population but not the other. We note that this is an alternative way to achieve genome-wide trans-ethnic genetic correlation of 0.80, as in our main simulations. In these simulations, S-LDXR yielded unbiased estimates of enrichment/depletion of squared trans-ethnic genetic correlation ($\lambda^2(C)$). We have updated the *Simulations* subsection of the *Results* section (see page 7, Figure S11, Table S12) and the *Methods* section (see page 23) to include these simulations.

(We did not perform simulations with *entirely* different causal variants between populations, because in this case r_g would be 0, and $\lambda^2(C)$ would be undefined.)

9. For GWAS, the true "causal" variants may not have been genotyped, so SNPs that may be in LD with the casual variant are used for assessing trans-ethnic genetic correlations instead of the true casual variants. How does using SNPs in LD with the causal variant impact the S-LDXR statistic when there are differential LD patterns in the causal region across populations? It would be helpful for the authors to provide some insight into this setting, which often occurs in GWAS.

The reviewer's question is related to Reviewer 1 Comment 1, which asked if results pertain to HapMap3 SNPs only (given that only HapMap3 SNPs are analyzed) and may thus be impacted by tagging of other SNPs. See part (a) of response to Reviewer 1 Comment 1. Also see part (a) of response to Reviewer 1 Comment 4.

S-LDXR analyzes summary statistics from HapMap3 SNPs with MAF > 5% in both populations (*regression SNPs*) together with reference panel LD from SNPs present in either population in 1000 Genomes (*reference SNPs*) to estimate enrichment/depletion (defined as a function of causal effect sizes) for *all* SNPs with MAF > 5% in both populations (*heritability SNPs*), accounting for tagging effects (analogous to S-LDSC; Finucane et al. 2015 *Nat Genet*, ref. 21). Thus, the results pertain to causal effects of *all* SNPs with MAF > 5% in both populations (*heritability SNPs*). We recognize that it is our responsibility to verify that not just the definition but also the actual estimates achieve this result. Thus, we simulated traits using SNPs present in either population in 1000 Genomes (including non-HapMap3 SNPs) as causal SNPs in our simulations, including simulations with annotation-dependent MAF-dependent architectures.

We have updated the *Introduction* section (see page 3), *Overview of methods* subsection of the *Results* section (see pages 4-5) and *Methods* section (see pages 18-19) to clarify this point.

Reviewer #3 (Remarks to the Author):

The authors describe a novel method to measure trans-ethnic squared genetic correlation, or a way to quantify the enrichment/depletion of similarity of causal effect sizes between populations. This new method can be useful in identifying annotations that may have increased population-differentiation of causal effect sizes, to hopefully downstream improve prediction and understanding of the underlying biology of traits. They provide simulations and the application of these methods to 30 traits comparing Europeans and Japanese summary statistics.

We thank Reviewer 3 for accurately summarizing our study, and for suggesting that our method can be useful.

In general, the manuscript would benefit from an expansion of potential uses for this novel method and interpretations of results when applied across traits. It is unclear as it currently stands why other investigators would adopt this method, especially in a trait-specific manner as the majority of analyses are conducted as a meta-analysis across all traits. This would require expansion in both the Introduction and Discussion sections.

The reviewer has raised several related questions/suggestions: (a) Is it of interest to apply S-LDXR in a trait-specific fashion; (b) How should results be interpreted when S-LDXR is applied to a meta-analysis across traits; and (c) The discussion of potential uses of S-LDXR should be expanded. We address each of these questions/suggestions in turn.

(a) Is it of interest to apply S-LDXR in a trait-specific fashion.

We thank the reviewer for the suggestion to explore trait-specific analyses in greater depth. Based on the reviewer suggestion, we have added new content (including a new Figure 5) on trait-specific analyses. Although statistically significant results were limited due to the reduced power of trait-specific analyses, we determined that SNPs around genes specifically expressed in pituitary and fibroblasts have population-specific causal effect sizes for BMI and height, respectively (see Figure 5, Table S21), suggesting that biological mechanisms for BMI and height may be different across East Asians and Europeans (also see response to Reviewer 3 Major Comment 2). We have updated the *Analysis of specifically expressed gene annotations* subsection of the *Results* section (see pages 10-11) to include this result.

Above and beyond assessing the statistically significant results, we conducted a comparison of BMI and height results for SNPs around specifically expressed genes in the 53 tissues (also see response to Reviewer 3 Major Comment 2). We determined that enrichment/depletion of squared trans-ethnic genetic correlation is similar for BMI and height across the 53 tissues, despite trait-specific depletion in pituitary and fibroblasts respectively (see Figure 5). We have updated the *Analysis of specifically expressed gene annotations* subsection of the *Results* section (see pages 10-11) to include this result.

We have also updated the *Introduction* section (see page 3) and *Discussion* section (see page 12) to discuss the motivation for assessing meta-analyzed vs. trait-specific results (also see response to Reviewer 3 Major comment 2).

(b) How should results be interpreted when S-LDXR is applied to a meta-analysis across traits.

Although biological processes differ across diseases and complex traits, patterns of functional enrichment/depletion are often similar (particularly for non-tissue-specific annotations), motivating previous studies to meta-analyze functional enrichment/depletion results across traits (e.g. S-LDSC; Finucane et al. 2015 *Nat Genet*, ref. 21). Therefore, we report most findings based on meta-analyses across the 31 traits. (We emphasize that we are meta-analyzing *enrichment/depletion*, not GWAS summary statistics.) Our interpretation of the meta-analyzed findings is that depletion of trans-ethnic genetic correlation in functional regions is likely attributable to stronger gene-environment interaction at functional regions impacted by positive selection.

We have updated the *Introduction* section (see page 3) and *Discussion* section (see page 12) to clarify these points.

(c) The discussion of potential uses of S-LDXR should be expanded.

We agree that additional discussion of the potential uses of S-LDXR is warranted. In addition to further discussion of trait-specific analyses (see (a) above), we have added additional discussion of applications to trans-ethnic PRS to the *Discussion* section (see pages 13-14). See our response to Reviewer 3 Major Comment 3.

Major Comments (Numbers added to each Major Comment)

1. The manuscript is currently written with much of the methods in the results section. This is understandable, as it is a methods development manuscript, but for this specific audience it should be rewritten with the methods in the results section simplified and the vast majority of this text in the methods section.

We have reduced the length of the *Overview of methods* subsection of the *Results* section by 35% (see pages 4-5), as recommended by the reviewer; this content has been moved to the *Methods* section (see pages 18-19). We found it challenging to reduce the length of the *Overview of methods* subsection any further, as certain quantities (e.g. enrichment/depletion of squared trans-ethnic genetic correlation) are described throughout the manuscript, making it a high priority to first define them.

2. All of the trait-related results are presented as a meta-analysis of all 30 diseases and complex traits. It would be great to have a section discussing trait- or trait group-specific trends. For example, do the two anthropometric traits (height, BMI) show different patterns, given that their heritability is much different? It would be beneficial to expand discussion of these trait-

specific trends to inform the reader of possible uses for this method in more trait focused projects.

We thank the reviewer for the suggestion to explore trait-specific analyses in greater depth. Based on the reviewer suggestion, we have added new content (including a new Figure 5) on trait-specific analyses. Although statistically significant results were limited due to the reduced power of trait-specific analyses, we determined that SNPs around genes specifically expressed in pituitary and fibroblasts have population-specific causal effect sizes for BMI and BMI (see Figure S5, Table S21), suggesting that biological mechanisms for BMI may be different across East Asians and Europeans (also see response to Reviewer 3 Introductory comments, paragraph 2). We have updated the *Analysis of specifically expressed gene annotations* subsection of the *Results* section (see pages 10-11) to include this result.

Above and beyond assessing the statistically significant results, we conducted a comparison of BMI and height results for SNPs around specifically expressed genes in the 53 tissues (also see response to Reviewer 3 Introductory comments, paragraph 2). We determined that enrichment/depletion of squared trans-ethnic genetic correlation is similar for BMI and height across the 53 tissues, despite trait-specific depletion in pituitary and fibroblasts respectively (see Figure 5). We have updated the *Analysis of specifically expressed gene annotations* subsection of the *Results* section (see pages 10-11) to include this result. We note that although S-LDXR results are different for height vs. BMI, enrichment/depletion of squared trans-ethnic genetic correlation is not expected to depend on heritability, as an increase in trait heritability would increase both the numerator (covariance) and denominator (variance) of trans-ethnic genetic correlation (Equation 2). We have updated the *Analysis of specifically expressed gene annotations* subsection of the *Results* section (see page 9) to clarify this point.

We have also updated the *Introduction* section (see page 3) and *Discussion* section (see page 12) to discuss the motivation for assessing meta-analyzed vs. trait-specific results.

3. A possible implication is cited as reweighting PRS from European population training data based on enrichment/depletion of the squared trans-ethnic genetic correlation. How would this practically be done in respect to directionality of effect weights? Would this just be downweighting SNPs that are depleted (therefore more likely to be different) and upweighting SNPs that are enriched (more likely to transfer)? And subsequently, would this improve performance or trend towards the null in terms of prediction if the larger effect sizes are in differentiated regions? It would be great to expand on this point in the discussion, given it is also mentioned in the introduction.

We agree that additional discussion of applications to trans-ethnic PRS is warranted. The reviewer is correct that, when computing PRS in a non-European population using effect size estimates from European training data, SNPs that are expected to be depleted for squared trans-ethnic genetic correlation based on their functional annotations should be downweighed and SNPs that are expected to be enriched for squared trans-ethnic genetic correlation based

on their functional annotations should be upweighted. For example, when applying LD-pruning + p-value thresholding methods to marginal effect sizes, SNPs in the latter category should be preferentially retained. Analogously, when applying more recent methods that estimate posterior mean causal effect sizes (including functionally informed methods), these estimates should subsequently be weighted according to the expected enrichment/depletion for squared trans-ethnic genetic correlation based on their functional annotations. We believe these approaches would improve prediction accuracy (by prioritizing SNPs that are more transferable across populations), even though the prioritized SNPs will tend to have slightly smaller causal effect sizes than the deprioritized SNPs (due to an inverse relationship between heritability enrichment and enrichment/depletion of squared trans-ethnic genetic correlation; see response to Reviewer 1 Comment 5).

We have updated the *Discussion* section (see pages 13-14) to clarify these points.

4. The authors note a limitation is that gene expression measurements were conducted in Europeans and may not be applicable to non-European groups. When looking at trans-ethnic correlations and annotation categories, which reference group do you pick for these annotations? These analyses were conducted in the European-derived reference datasets. Would you need to do two analyses, one in European and one in Asian reference sets, and then compare/contrast?

The reviewer is correct that our manuscript stated that the specifically expressed gene (SEG) annotations analyzed in our study were defined primarily based on gene expression measurements in Europeans. In detail, we used the SEG annotations from Finucane et al. 2018 *Nat Genet* (ref. 24), which were derived from all GTEx samples, which include 84.6% European samples, 12.9% African American samples, and 1.3% Asian samples. We have updated the *Discussion* section (see page 14) to clarify that these samples are predominantly but not exclusively European.

We agree with the reviewer that the logical alternative analysis for comparison purposes would be to define SEG annotations based on gene expression measurements in East Asians. We have updated the *Discussion* section (see page 14-15) to clarify this point.

We hypothesize that results based on SEG annotations defined in Europeans vs. SEG annotations defined in East Asians would likely be similar, because heritability enrichments of functional annotations (ascertained predominantly in Europeans) are very similar across continental populations (see Kichaev and Pasaniuc 2015 *Am J Hum Genet*, ref. 31; Kanai et al. 2018 *Nat Genet*, ref. 20; Figure 2, 3, Table S14, S17 of our manuscript, cited on page 28 and page 29). We have updated the *Discussion* section (see pages 14-15) to clarify this point.

5. There is currently not a description of the summary statistics used for trait-specific analyses. For example, were these summary statistics genome-wide on a genotyping array or after imputation to 1000Genomes or another reference panel? Besides MAF cut-offs, were there any other thresholds used to select inclusion? As many PRS use a thresholded approach, how would

this affect the weighting if the depletion/enrichment were limited to SNPs above a certain p-value threshold, which would mean larger effect sizes given their common frequencies of MAF>5%?

The reviewer has raised two related questions/suggestions: (a) A more detailed description of the summary statistic data sets is warranted; and (b) Do thresholds used to determine the set of SNPs analyzed impact PRS strategies?

(a) A more detailed description of the summary statistic data sets is warranted.

We agree that a more detailed description of the summary statistic data sets is warranted, beyond what was previously provided in the *Methods* section and Table S13.

All summary statistics were based on imputation to an appropriate LD reference panel (e.g. Haplotype Reference Consortium for UK Biobank, 1000 Genomes for Biobank Japan).

We analyzed summary statistic data for HapMap3 SNPs with MAF > 5% in both populations (*regression SNPs*), in conjunction with reference panel LD from SNPs present in either population in the 1000 Genomes Project (*reference SNPs*) (analogous to S-LDSC; Finucane et al. 2015 *Nat Genet*, ref. 21) (see part (a) of response to Reviewer 1 Comment 1, part (a) of response to Reviewer 1 Comment 4, response to Reviewer 2 Comment 9). HapMap3 SNPs consist predominantly of common, well-imputed SNPs, such that study-specific MAF and imputation accuracy thresholds are inconsequential. No other criteria were used to select the summary statistic SNPs that we analyzed.

We have updated the *Methods* section (see page 18 and page 24) to clarify these points. All GWAS summary statistics that we analyzed are publicly available (see Table S13), and we refer the readers to the corresponding publications (cited in Table S13) for a detailed description of how each GWAS was performed.

(b) Do thresholds used to determine the set of SNPs analyzed impact PRS strategies?

As noted above, we analyzed summary statistic data for HapMap3 SNPs (*regression SNPs*), which consist predominantly of common, well-imputed SNPs, such that study-specific MAF and imputation accuracy thresholds are inconsequential. On the other hand, when constructing PRS, it might be advantageous to include non-HapMap3 SNPs.

The reviewer mentioned the (LD-pruning +) p-value thresholding approach often used to construct PRS. However, this type of threshold is distinct from the threshold used to determine the set of SNPs analyzed, as we did not restrict our S-LDXR analyses based on a p-value threshold, nor did we construct a genomic annotation based on a p-value threshold. We do not recommend constructing a genomic annotation based on a p-value threshold, which would be confounded by LD and subject to overfitting.

It is possible that G×E is stronger at SNPs passing a p-value threshold (and stronger at SNPs with larger causal effects), implying depletion of trans-ethnic genetic correlation for such SNPs.

Prioritizing SNPs for trans-ethnic PRS should account for both the strength of association and trans-ethnic genetic correlation (see response to Reviewer 3 Major comment 3). We have updated the *Discussion* section (see pages 13-14) to clarify these points.

6. Top 5% of simulations with highest standard errors were discarded to assess unbiasedness of estimator. However, the authors note that in the analysis of real traits, these estimates would contribute very little to the meta-analysis across traits. Does this mean this method should only be used across multiple traits and not for a few traits on their own?

In the case of a meta-analysis across traits, the reviewer is correct that estimates with very high standard errors would contribute very little to the meta-analysis, as previously noted in the *Simulations* subsection of the *Results* section (p.6).

In the case of trait-specific analyses, in the types of scenarios that occurred in our simulations, S-LDXR would produce trait-specific estimates with very high standard errors, which would be interpreted as being inconclusive. We believe that the possibility of producing inconclusive estimates should not preclude trait-specific analyses using S-LDXR. (Indeed, statistically significant results in our trait-specific analyses were broadly limited due to the reduced power of trait-specific analyses; see response to Reviewer 3, Introductory comments paragraph 2 and response to Reviewer 3, Major comment 2.) We have updated the *Simulations* subsection of the *Results* section (p.6) to clarify this point.

7. The method compares the similarity versus dissimilarity of effect sizes across different populations. However, it would also be useful to include a discussion of effect size magnitudes, which ties into the heritability and the number of SNPs considered.

We agree with the reviewer that more discussion of the relationship between enrichment of heritability and enrichment/depletion of squared trans-ethnic genetic correlation is warranted (also see response to Reviewer #1 Comment 5).

We observed a general trend that annotations enriched for heritability show depletions of squared trans-ethnic genetic correlation. However, we believe that the fundamental cause of depletion of trans-ethnic genetic correlation is gene-environment interaction (G×E), not heritability enrichment. Therefore, an annotation enriched for SNPs exhibiting G×E may show depletion of squared trans-ethnic genetic correlation, even if the annotation is depleted of heritability. Conversely, an annotation depleted of SNPs exhibiting G×E may show enrichment of squared trans-ethnic genetic correlation, even if the annotation is enriched for heritability. We discuss these points in the *Discussion* section (see page 12).

Minor Comments

- Figures S4-6: The legends for these figures are confusing. For each of these figures, the legends only refer to panels a and b (S4), c and d (S5), or e (S6). The legends should be expanded to explicitly state the differences between all panels.

We thank the reviewer for flagging the incomplete captions for Figures S4-S6.

In Figures S4-S6, each panel corresponds to simulation results obtained with different shrinkage parameters (in Figure S4 and S5, α=0.0, 0.25, 0.75, and 1.0 in panel a, b, c, and d, respectively; in Figure S6, α=0.0, 0.25, 0.5, 0.75, and 1.0 in panel a, b, c, d, and e, respectively). We have updated the captions of Figure S4-S6 accordingly.

- Figure S7: What are the blue and red filled dots symbolizing?

We thank the reviewer for flagging the inadequate description of blue and red filled dots in the caption of Figure S7.

In Figure S7 (now Figure S12), red dots represent diseases/traits with estimated genome-wide trans-ethnic genetic correlation (r_g) significantly less than 1 (one-tailed $p < 0.05/53$); blue dots represent traits for which estimated r_g is not significantly less than 1. We have updated the caption of this figure (now Figure S12) to clarify the interpretation of blue and red filled dots.

Reviewer #1 (Remarks to the Author):

I would like to thank the authors for responding each of my questions in detail. I have the following additional comments/questions based on their revised manuscript.

1. They performed additional analysis on SCZ and found a high genetic correlation between EUR and EAS populations. Because SCZ is believed to be under relatively high negative selection, they use this result as a piece of evidence to support their hypothesis that positive selection contributed most to the population-specific causal effects. While their explanation is plausible, an alternative possible reason for the high rg could be due to the ignoring of SNPs with MAF < 5%. Negative selection would have largest impact on the variants with low frequency, so rg may be actually low if they have included those SNPs in the analysis. In addition, SCZ has the smallest sample size in both populations for which the power to detect a rg different from 1 may be compromised given their shrinkage parameter. I think these may worth to be noted in discussion.

2. Background selection statistic seems to be the key annotation in their analysis, as almost all of the other annotations that shows depletion in lamba_C^2 are correlated with it, including average LLD, CpG content, recombination rate, binary functional annotations and tissue specifically expressed gene annotations. The authors demonstrate by simulations that their method is nearly unbiased to the null model. However, it seems there is systematic bias, although to a small magnitude, to the background selection statistic (Figure 1a), in a similar pattern to what observed in the real trait analysis (Figure 2b). Another annotation that appears to be influential, average LLD, which is moderately correlated with background selection statistic, also shows a systematic bias (in a pattern coincide with the real trait result) when a MAF-dependent architecture is simulated (Figure S9), which is likely to be true in reality. Although these biases are not large, I am worried that these small systematic biases would increase with the increase of sample size, and ultimately give false positives of enrichment/depletion. I think it worth to check that by simulating a GWAS sample as large as the real data sets, since the most important conclusions of this paper rely heavily on the result of background selection statistic annotation.

3. Table S13 shows a few traits with remarkably low rg. The authors attributed the low rg in Major Depressive Disorder to the discrepancy in diagnose criterion. Do they have an explanation to other traits that are well defined but have substantially lower rg, such as Age at menopause (rg=0.567), Basophil counts (rg=0.427) and LDL (rg=0.662)?

Reviewer #2 (Remarks to the Author):

The authors have adequately addressed my comments, and I find the revised manuscript to be much improved. The results presented in this paper illustrating that there are more population-specific causal effect sizes in functionally important regions is quite compelling. I think this work will be of interest to a broad range of genetic researchers, particularly those involved in complex trait mapping across diverse populations as well as population geneticists.

Reviewer #3 (Remarks to the Author):

The authors have addressed all of my questions/comments satisfactorily. The following suggestions may improve the readability and impact of the paper, but are not necessary for acceptance. Great job!

1. The limitations of the study are listed in the discussion. The way that they are structured right now does not flow very well and would benefit from being condensed and structured into themes, such as the 6th (MAF>5%) and 7th (population-specific variants) referring to allele frequency limitations.

2. The authors note that a major limitation is restricting analyses to East Asian and European populations, given the unavailability of other large-scale GWAS summary statistics. Would they expect results to change, given a different number of variants would be included with an African and European comparison, given different allele frequencies and LD structures comparing the larger (EUR) to smaller (AFR) datasets?
3. There are several run-on sentences that could either be simplified or shortened (Page 8, lines 236-242, for example. There's also an extra period at the end of the sentence after the reference).
4. Restricting the analyses to MAF>5% in both populations may lead to a selection bias in the results, in that the set of SNPs that are above 5% in East Asian populations are much smaller than above 5% in an African population given the SFS of East Asian groups versus European versus African. It would be great to see some discussion of how results may differ if EUR were compared to AFR in this regard.
5. An expansion on how this method can help trans-ethnic meta-analysis (such as adapting MTAG) would be useful.
6. The second point in the discussion for uses of this new method mentions improved trans-ethnic fine-mapping, moving beyond the assumption that causal variants are shared between populations. However, it is unclear how this conclusion is drawn from your results, given the high proportion of correlation between EUR and EAS results for many of the outcomes. For many of these statements in the discussion, it would be beneficial to expand on how or why these applications would be useful.

Response to reviewers for NCOMMS-19-37936-T (Shi et al.)

Reviewer #1 (Remarks to the Author):

I would like to thank the authors for responding each of my questions in detail. I have the following additional comments/questions based on their revised manuscript.

We thank Reviewer 1 for acknowledging our detailed responses.

1. They performed additional analysis on SCZ and found a high genetic correlation between EUR and EAS populations. Because SCZ is believed to be under relatively high negative selection, they use this result as a piece of evidence to support their hypothesis that positive selection contributed most to the population-specific causal effects. While their explanation is plausible, an alternative possible reason for the high rg could be due to the ignoring of SNPs with MAF < 5%. Negative selection would have largest impact on the variants with low frequency, so rg may be actually low if they have included those SNPs in the analysis. In addition, SCZ has the smallest sample size in both populations for which the power to detect a rg different from 1 may be compromised given their shrinkage parameter. I think these may worth to be noted in discussion.

The reviewer has raised two separate concerns: (i) exclusion of SNPs with MAF < 5% may bias trans-ethnic genetic correlation estimates for SCZ, and (ii) estimates of trans-ethnic genetic correlation for SCZ may be compromised by our shrinkage estimator due to low sample sizes for SCZ. We response to each of these concerns in turn:

(i) exclusion of SNPs with MAF < 5% may bias trans-ethnic genetic correlation estimates for SCZ.

The reviewer is correct that we analyze regression SNPs with MAF > 5% in both populations (to draw inferences about causal effects of heritability SNPs with MAF > 5% in both populations). As previously noted in the Discussion section, this is a limitation of our analyses, necessitated by the lack of a large LD reference panel in East Asians. However, we believe that our estimates of a trans-ethnic genetic correlation parameter *defined based on MAF > 5% SNPs* are robust, and that a comparison of this parameter for SCZ vs. other traits can be informative.

The reviewer is also correct that negative selection has the largest impact on variants with low frequency (Zeng et al. 2018 Nat Genet; ref. 26), such that analyses of low-frequency SNPs would be particularly informative for drawing inferences about the action of negative selection. However, negative selection also impacts common variant architectures (Gazal et al. 2017 Nat Genet; ref. 22), such that analyses of common variants can also be informative for drawing inferences about the action of negative selection.

We have expanded our discussion of SCZ and negative selection in the *Analysis of specifically expressed gene annotations* subsection of the Results section (page 11), and our discussion of low-frequency SNPs in the Discussion section (page 16), to clarify these points.

(ii) estimates of trans-ethnic genetic correlation for SCZ may be compromised by our shrinkage estimator due to low sample sizes for SCZ.

S-LDXR does not use the shrinkage estimator when estimating *genome-wide* trans-ethnic genetic correlation. We believe that estimates of genome-wide trans-ethnic genetic correlation for SCZ are robust (with well-calibrated standard errors), as shown by our simulations, and that our estimate for SCZ of 0.95 (s.e. 0.04) (for MAF > 5% SNPs) is clearly closer to 1 than the average of 0.85 (s.e. 0.01) across traits. We have updated the *Simulations* subsection of the Results section (page 5), our discussion of SCZ and negative selection in the *Analysis of specifically expressed gene annotations* subsection of the Results section (page 11), and the *S-LDXR shrinkage estimator* subsection of the Methods section (page 23), to clarify these points.

2. Background selection statistic seems to be the key annotation in their analysis, as almost all of the other annotations that shows depletion in lamba_C^2 are correlated with it, including average LLD, CpG content, recombination rate, binary functional annotations and tissue specifically expressed gene annotations. The authors demonstrate by simulations that their method is nearly unbiased to the null model. However, it seems there is systematic bias, although to a small magnitude, to the background selection statistic (Figure 1a), in a similar pattern to what observed in the real trait analysis (Figure 2b). Another annotation that appears to be influential, average LLD, which is moderately correlated with background selection statistic, also shows a systematic bias (in a pattern coincide with the real trait result) when a MAF-dependent architecture is simulated (Figure S9), which is likely to be true in reality. Although these biases are not large, I am worried that these small systematic biases would increase with the increase of sample size, and ultimately give false positives of enrichment/depletion. I think it worth to check that by simulating a GWAS sample as large as the real data sets, since the most important conclusions of this paper rely heavily on the result of background selection statistic annotation.

The reviewer is correct that estimates for the top quintile of background selection statistic show a small systematic bias in null simulations: -0.023 (s.e. 0.003) in Figure 1a, -0.013 (s.e. 0.008) in annotation-dependent MAF-dependent architecture simulations of Figure S9a, where -0.023 and -0.013 are smaller in magnitude but in the same direction as -0.18 (s.e. 0.01) for real traits in Figure 2b. Similarly, average LLD also shows a small systematic bias in null simulations: -0.022 (s.e. 0.005) in Figure 1a, -0.037 (s.e. 0.008) in annotation-dependent MAF-dependent architecture simulations of Figure S9a, where -0.022 and -0.037 are smaller in magnitude but in the same direction as -0.13 (s.e. 0.03) for real traits in Figure 2b.

The reviewer is also correct that our HAPGEN2 simulated sample sizes (N_{EAS}=18K, N_{EUR}=37K) are smaller than sample sizes for real traits (average N_{EAS}=90K, N_{EUR}=267K). The reviewer suggested that we perform simulations at much larger sample sizes (matching sample sizes for real traits), to rule out the possibility that small systematic biases might increase with sample size.

We agree that performing simulations at much larger sample sizes, if feasible, would be ideal. However, we determined that it was not computationally feasible to analyze much larger simulated sample sizes, due to computational challenges in applying the relatedness filter to the HAPGEN2 simulated samples: the running time and memory cost of the relatedness filter scales with the square of the number of samples, and the relatedness filter removed roughly ~64% of the 100,000 HAPGEN2 simulated samples in each population—a proportion that is expected to grow larger with larger simulation sample size, due to the limited number of EAS and EUR haplotypes provided as input to HAPGEN2. We instead performed 3 new simulations, *decreasing* or *increasing* the reference panel size or *decreasing* the HAPGEN2 simulated GWAS sample size, based on the baseline-LD-X model (analogous to Figure 1). We also performed 3 corresponding new simulations, based on the model with annotation-dependent MAF-dependent architectures (analogous to Figure S9).

First, we *decreased* or *increased* the size of the S-LDXR reference panel from 500 samples to 250 or 1,000 samples, in order to probe the possible impact of mismatch between in-sample LD and reference panel LD. We determined that the small systematic biases in null simulations of continuous-valued annotations were on the same order of magnitude as for 500 reference samples (Figures S12, S13 and Table 13 for 250 reference samples; Figures S14, S15 and Table S14 for 1,000 reference samples). For example, for the top quintile of background selection statistic (simulations using baseline-LD-X model), the bias of -0.023 (s.e. 0.003) in Figure 1a changed to +0.01 (s.e. 0.004) with 250 reference samples and also +0.01 (s.e. 0.004) with 1,000 reference samples. For the top quintile of average LLD (simulations using annotation-dependent MAF-dependent architecture), the bias of -0.037 (s.e. 0.008) in Figure S9a changed to -0.052 (s.e. 0.008) with 250 reference samples and -0.01 (s.e. 0.009) with 1,000 reference samples.

Second, we *decreased* the HAPGEN2 simulated GWAS sample sizes from N_{EAS}=18K, N_{EUR}=37K to N_{EAS}=9K, N_{EUR}=18K. We determined that the small systematic biases in null simulations of continuous-valued annotations were generally on the same order of magnitude as for N_{EAS}=18K, N_{EUR}=37K (Figure S16, S17 and Table S15). For example, for the top quintile of background selection statistic (simulations using baseline-LD-X model), the bias of -0.023 (s.e. 0.003) in Figure 1a changed to +0.02 (s.e. 0.007). For the top quintile of average LLD (simulations using annotation-dependent MAF-dependent architecture), the bias of -0.037 (s.e. 0.008) in Figure S9a changed to +0.01 (s.e. 0.01). However, the estimates in these simulations were generally less stable (higher standard error) and sometimes subject to larger biases (for both binary and continuously valued annotations), likely because our analytical bias correction starts to break

down when the GWAS has low power. For example, for the 5' UTR annotation (simulations using baseline-LD-X model), the bias of -0.02 (s.e. 0.01) in Figure 1b changed to +0.09 (s.e. 0.03).

Overall, these analyses do not provide a reason to believe that the small biases that we observed in some of our null simulations of continuously valued annotations would substantially increase at larger GWAS sample sizes. We have updated the *Simulations* subsection of the Results section (page 7, citing Figures S12-S17 and Tables S13-S15), and the *Simulations* subsection of the *Methods* section (page 25), to report these new experiments. Recognizing that average LLD is the annotation most susceptible to bias across all of our simulations, we have also added an appropriate caveat to our average LLD results (which are not central to our overall conclusions) in the *Analysis of baseline-LD-X model annotations across 31 diseases and complex traits subsection* of the *Results* section (page 8).

We further note that our estimates are *unbiased* in null simulations of binary annotations (Figure 1b and Figure S8), implying that our results on real traits for binary annotations (Figure 3a, corroborating results on real traits for continuous-valued annotations in Figure 2b) are extremely robust. We have updated the *Discussion* section (page 15) to carefully note both the small biases in null simulations of continuous-valued annotations, and the lack of bias in null simulations of binary annotations.

3. Table S13 shows a few traits with remarkably low rg. The authors attributed the low rg in Major Depressive Disorder to the discrepancy in diagnose criterion. Do they have an explanation to other traits that are well defined but have substantially lower rg, such as Age at menopause (rg=0.567), Basophil counts (rg=0.427) and LDL (rg=0.662)?

The reviewer is correct that we have estimated low genome-wide trans-ethnic genetic correlations—with low standard errors—for Age at Menopause (r_g=0.57 (s.e. 0.09)), Basophil Count (r_g=0.43 (s.e. 0.06)), and LDL (r_g=0.66 (s.e. 0.11)). We hypothesize that the low genome-wide trans-ethnic genetic correlations for these traits is due to pervasive gene-environment interaction across the genome. We have updated the *Analysis of baseline-LD-X model annotations across 31 diseases and complex traits* subsection of the Results section to clarify this point (page 8).

Reviewer #2 (Remarks to the Author):

The authors have adequately addressed my comments, and I find the revised manuscript to be much improved. The results presented in this paper illustrating that there are more population-specific causal effect sizes in functionally important regions is quite compelling. I think this work will be of interest to a broad range of genetic researchers, particularly those involved in complex trait mapping across diverse populations as well as population geneticists.

We thank Reviewer 2 for suggesting that the revised manuscript is much improved, and that our results are quite compelling and of interest to a broad range of genetic researchers.

Reviewer #3 (Remarks to the Author):

The authors have addressed all of my questions/comments satisfactorily. The following suggestions may improve the readability and impact of the paper, but are not necessary for acceptance. Great job!

We thank Reviewer 3 for indicating that the questions/comments have been addressed. The additional reviewer suggestions for improving readability and impact are addressed below.

1. The limitations of the study are listed in the discussion. The way that they are structured right now does not flow very well and would benefit from being condensed and structured into themes, such as the 6th (MAF>5%) and 7th (population-specific variants) referring to allele frequency limitations.

We thank the reviewer for the suggestion to restructure the presentation of the limitations in the *Discussion* section. Based on this suggestion, we have split the limitations part of the *Discussion* section (formerly a single paragraph) into two separate paragraphs; the first paragraph focuses on the limitations of the S-LDXR method (pages 15-16), and the second paragraph focuses on the limitations of our analysis of real traits (pages 16-17).

We elected to retain the limitation of restricting to MAF > 5% SNPs and the limitation of not considering population-specific variants as separate limitations, as restricting to MAF > 5% SNPs is a function of the available data (specifically, the lack of a large LD reference panel in East Asians) whereas defining trans-ethnic genetic correlation for population-specific variants is a more fundamental challenge, as we now clarify in the Discussion section (page 16).

2. The authors note that a major limitation is restricting analyses to East Asian and European populations, given the unavailability of other large-scale GWAS summary statistics. Would they expect results to change, given a different number of variants would be included with an African and European comparison, given different allele frequencies and LD structures comparing the larger (EUR) to smaller (AFR) datasets?

The reviewer makes a good point that a different set of variants (with different MAF and LD patterns) would be included when analyzing data from African vs. European populations. However, we expect that estimates of genome-wide genetic correlation and enrichment of stratified squared trans-ethnic genetic correlation would be broadly similar for African vs. European as compared to East Asian vs. European if patterns of gene-environment interaction (G×E) in the African population are similar to those in the East Asian population. Since we hypothesize that G×E is the fundamental factor impacting trans-ethnic genetic correlation, we do not expect a different set of variants (with different MAF and LD patterns) to lead to

differences in trans-ethnic genetic correlation. We have updated the *Discussion* section (page 16-17) to clarify this point. (Also see response to Reviewer #3 Comment 4.)

3. There are several run-on sentences that could either be simplified or shortened (Page 8, lines 236-242, for example. There's also an extra period at the end of the sentence after the reference).

We have updated the 2 sentences that the reviewer is referring to (page 9).

4. Restricting the analyses to MAF>5% in both populations may lead to a selection bias in the results, in that the set of SNPs that are above 5% in East Asian populations are much smaller than above 5% in an African population given the SFS of East Asian groups versus European versus African. It would be great to see some discussion of how results may differ if EUR were compared to AFR in this regard.

The reviewer makes a good point that a different set of variants (with different MAF and LD patterns) would be included when analyzing data from African vs. European populations. However, we expect that estimates of genome-wide genetic correlation and enrichment of stratified squared trans-ethnic genetic correlation would be broadly similar for African vs. European as compared to East Asian vs. European if patterns of gene-environment interaction (G×E) in the African population are similar to those in the East Asian population. Since we hypothesize that G×E is the fundamental factor impacting trans-ethnic genetic correlation, we do not expect a different set of variants (with different MAF and LD patterns) to lead to differences in trans-ethnic genetic correlation. We have updated the *Discussion* section (page 16) to clarify this point. (Also see response to Reviewer #3 Comment 2.)

5. An expansion on how this method can help trans-ethnic meta-analysis (such as adapting MTAG) would be useful.

We thank the reviewer for the suggestion to expand on the point that modeling population-specific architectures may increase power in trans-ethnic meta-analysis, e.g. by adapting MTAG (Turley et al. 2018 Nat Genet; ref. 70) to two populations (instead of two traits). We have updated the Discussion section (page 15) to expand on this point.

6. The second point in the discussion for uses of this new method mentions improved trans-ethnic fine-mapping, moving beyond the assumption that causal variants are shared between populations. However, it is unclear how this conclusion is drawn from your results, given the high proportion of correlation between EUR and EAS results for many of the outcomes. For

many of these statements in the discussion, it would be beneficial to expand on how or why these applications would be useful.

We thank the reviewer for the suggestion to expand on the point about improving trans-ethnic fine-mapping. We have updated the Discussion section (page 15) to expand on this point.

Reviewer #1 (Remarks to the Author):

The authors have adequately addressed my questions. I would like to congratulate the authors on completing this important work!
